# How Digital Images Are Transforming Chemical Education: A Review of Laboratory-Based Applications

**DOI:** 10.1021/acsomega.5c05125

**Published:** 2025-07-21

**Authors:** Jennifer García Ramos, Roberto Silva de Souza Júnior, Endler Marcel Borges

**Affiliations:** † Department of Chemistry, 311308 Purdue University, West Lafayette, Indiana 47907, United States; ‡ Departamento de Química, Fundação Universidade Regional de Blumenau, FURB, Campus 1, Rua Antônio da Veiga, 140, Victor Konder, 89012-900 Blumenau-SC, Brazil

## Abstract

This review explores the transformative role of digital imaging technologies—including smartphones, webcams, scanners, and digital cameras—in contemporary chemical education and laboratory-based analysis. These tools have emerged as accessible and cost-effective alternatives to traditional spectrophotometric instruments, enabling the capture and quantification of color changes in chemical reactions through RGB value extraction. The review presents a comprehensive overview of the technical principles underlying digital image acquisition, addressing factors such as lighting conditions, device variability, color spaces, and image formats, and examines their impact on analytical accuracy and reproducibility. A wide array of laboratory experiments is discussed, spanning analytical and physical chemistry, with applications in colorimetric assays, fluorescence, flame emission, titrations, and chemical equilibrium studies. Digital imaging has been successfully applied to quantify various analytes, including food dyes, proteins, pharmaceuticals, cations, and anions. The review also emphasizes the pedagogical benefits of these approaches, particularly in remote and resource-limited settings where students can perform meaningful scientific investigations using their own devices. The integration of digital imaging into laboratory instruction promotes student engagement, autonomy, and inquiry-based learning. Its widespread adoption was further accelerated by the COVID-19 pandemic, which demonstrated the feasibility of at-home experimentation. As imaging technologies continue to advance, their potential to democratize access to scientific tools and enhance chemical education is expected to expand, fostering a more inclusive, innovative, and effective approach to laboratory science.

## Graphical abstract

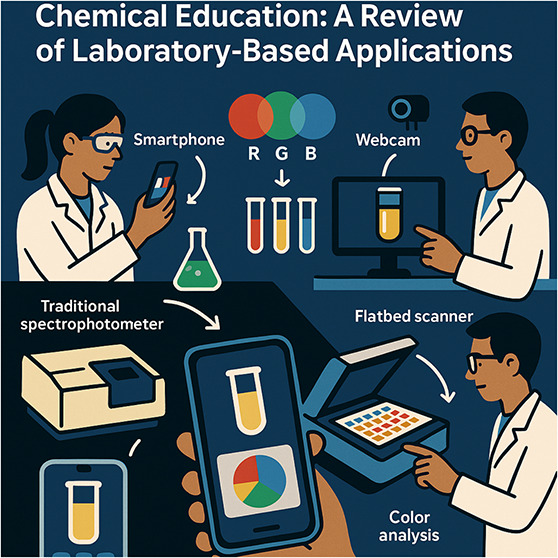

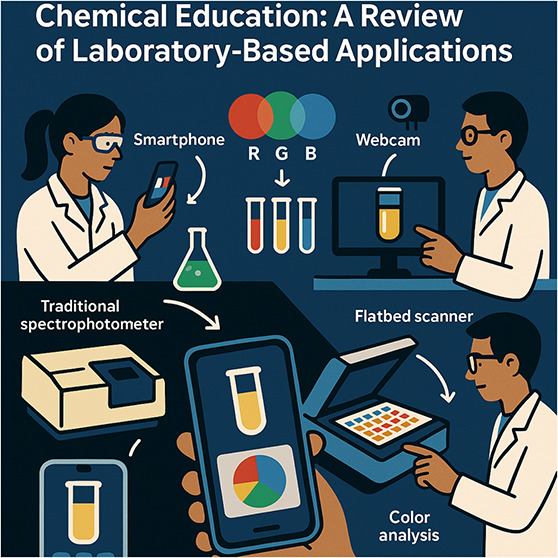

## Introduction

Digital images acquired using smartphones, cameras, webcams, and scanners have been widely utilized for a variety of analytical applications due to their advantages, such as portability, low energy demand, cost-effectiveness and user-friendliness.^1^ In contemporary scientific research and education, there is a growing interest in chemical analysis methods centered around color changes, a phenomenon meticulously recorded using advanced imaging devices.^2–4^ These methods encompass a wide array of tools, ranging from traditional cameras and scanners to the ubiquitous presence and use of smartphones. The smartphone emerged as an essential asset, offering convenient and effective alternatives for colorimetric analysis in various applications.^5^

Digital imaging technology has spearheaded a significant shift in the field of quantitative analysis. Researchers have extensively explored the potential of images captured by multimedia devices, showcasing their effectiveness in replacing traditional spectrophotometric methods. The scientific community has witnessed a surge in comprehensive reviews comparing the capabilities of spectrophotometers with these versatile imaging devices.^6–9^

## DIgital Image Technologies: Applications and Trends in Different Fields

In 2015, Capitán-Vallvey et al.^8^ published a comprehensive review on the use of digital images acquired with smartphones, cameras, webcams, and digital cameras as detectors in analytical applications. While the majority of studies focused on the use of smartphones for colorimetric assays, the review also covered other optical detection methods, including fluorescence, chemiluminescence, bioluminescence, and photoluminescence. The authors highlighted a dramatic rise in the number of publications utilizing these devices between 1995 and 2015, reflecting their growing importance in analytical chemistry.

Fernandes et al.^7^ reviewed the use of smartphones in colorimetric assays, highlighting a steady increase in the number of publications from 2015 to 2020. Fan et al.^9^ also conducted a review focused on colorimetric assays using smartphones as detectors, with particular emphasis on the various color spaces employed for analysis, including RGB, CMY/CMYK, HSB/HSL, and L*a*b*.

Soares et al.^1^ emphasized that digital images can be captured using a range of devices, including scanners, digital cameras, and webcams. However, smartphone cameras are generally preferred in analytical applications due to their portability, accessibility, and significantly lower cost compared to traditional UV–vis spectrophotometers. The authors detailed the use of smartphones as detectors across multiple fields, including environmental monitoring, food analysis, fuel quality assessment, pharmaceutical testing, and clinical diagnostics.

Rezazadeh et al.^5^ explored the use of smartphones as optical detectors in analytical applications. Roda et al.^10^ provided a comprehensive review of smartphones functioning as biosensors in point-of-care and point-of-need platforms, with applications in healthcare, food safety, environmental monitoring, and biosecurity. Nelis et al.^11^ focused specifically on food safety, presenting a review of smartphone-based optical assays that ranged from peer-reviewed research to commercially available colorimetric tests. Phuong Le et al.^12^ and Xie et al.^13^ examined the development of smartphone-assisted molecularly imprinted polymer (MIP) optical biosensors, discussing advances in sensor design, molecular recognition, signal transduction mechanisms, and their promising applications for on-site detection of food contaminants.

During COVID-19 pandemic, several naked-eye SARS-CoV-2 test kits have been developed as home tests, Li et al.^14^ had reviewed how smartphones can convert various signals into digital information, improving the sensitivity and accuracy of these home tests. Other reviews also described the use of smartphones as biosensor during the pandemic.^15,16^

Xing et al.^17^ reviewed how smartphones could be used as detectors in microfluidic sensing and summarized the detection routes, including colorimetric, fluorescent, electrochemical and chemiluminescent detection.

Gopal and Muthu^18^ reviewed how smartphones can be used as detector for portable tests for food adulteration. Geballa-Koukoula et al.^19^ and Ross et al.^20^ reviewed the use of smartphones as detectors in conjunction with biosensors. Christodouleas et al.^21^ and Wang et al.^22^ reviewed smartphones point-of-care technologies and detection of infectious diseases, respectively. Those reviews covered topics such as colorimetric, electrochemical, and biochemiluminescent detection using smartphones.

Li et al.^23^ reviewed how smartphones integrated with nanomaterials were used for the detection for heavy metal ions. Mousavizadegan et al.^24^ described the use of smartphones in point-of-care and food safety. The review was focused on signal processing and chemometric approach. Vu et al.^25^ described how smartphones can be used in point of care diagnostics.

Kovarik et al.^26^ have emphasized the growing integration of homemade spectrophotometers and imaging devices in chemical education. More recently, da Silva Oliveira et al.^27^ reported an increasing number of laboratory experiments employing digital imaging techniques over the past decade. The versatility and effectiveness of digital images in quantitative analysis are well established, and their continued adoption in educational settings holds great potential to enhance student learning and stimulate innovation. This article aims to provide an overview of laboratory experiments that utilize digital imaging technology, highlighting their applications, benefits, and pedagogical value.

## Color Spaces

A color space is a defined range of colors visible to the human eye or to devices. There are several color models/color spaces, including RGB, CMYK, HSV/HSL, CIE XYZ, L*a*b*, and YUV, which are frequently utilized in digital image colorimetry.^1^ The most commonly used color space is RGB, where each color component (Red, Green, and Blue) is assigned to one of the three orthogonal coordinate axes in a 3D space. For instance, in the RGB space, white is represented as (255,255,255), black as (0,0,0), red as (255,0,0), green as (0,255,0), blue as (0,0,255), magenta as (255,0,255), yellow as (255,255,0), and cyan as (0,255,255).^28,29^

Color perception arises from the wavelengths of light that an object or solution reflects. For example, a blue t-shirt appears blue because it absorbs red light while reflecting blue wavelengths. In chemical solutions, the color perceived by the human eye is typically the complement of the color that is most strongly absorbed (Figure 1).^30^ For instance, a permanganate solution exhibits a maximum absorbance (λ_max_) in the green region (525–545 nm), resulting in a red-violet appearance. The human eye is more sensitive to blue and red light than to orange or violet, which helps explain why the sky appears blue rather than violet: although violet light is more efficiently scattered via Rayleigh scattering, our greater sensitivity to blue makes it the dominant color perceived.^31^

**Figure 1:**
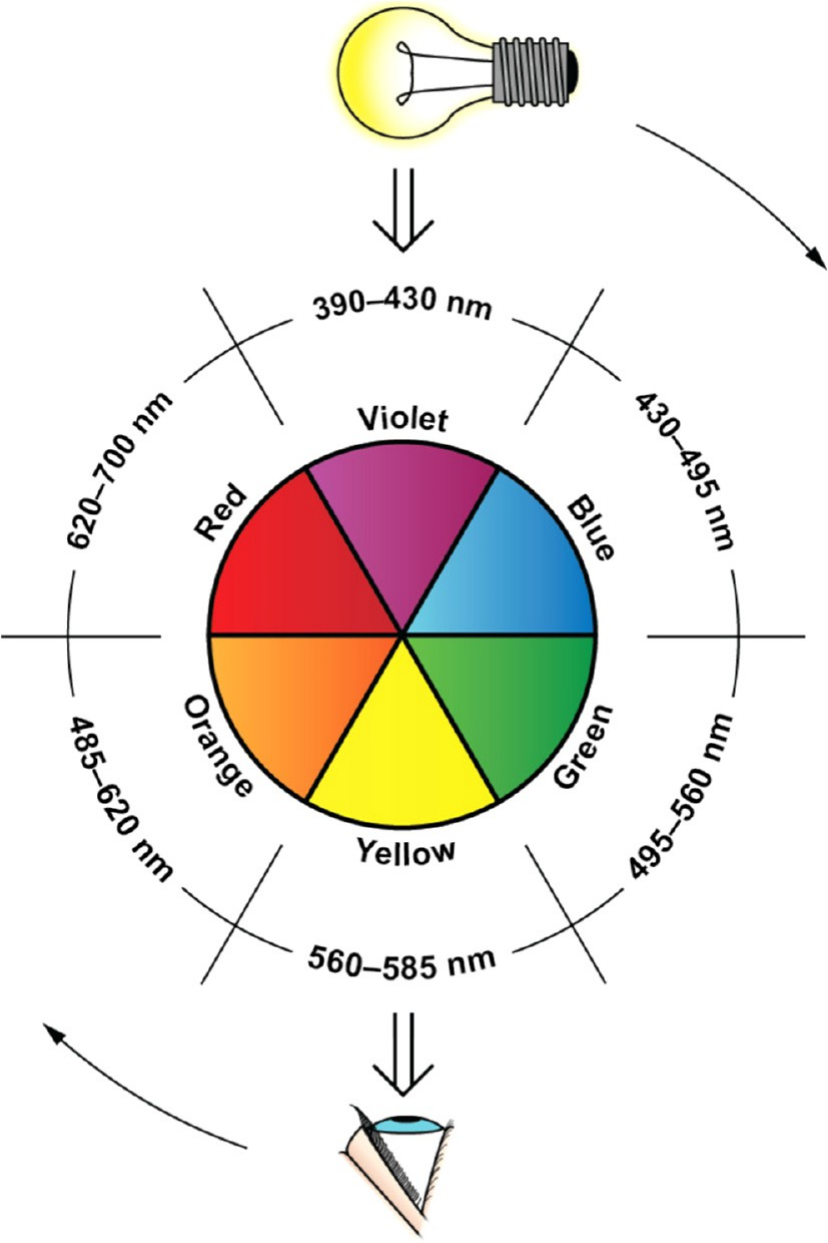
Color wheel showing the approximate relationship between the color or wavelength of light absorbed and the color observed. The light bulb and eye rotate synchronously around the wheel. As drawn, the figure shows that a solution that absorbs violet light will appear yellow to the eye (Figure reproduced from Algar et al.^30^).

This concept extends to other colored complexes, such as Fe­(SCN)_2_^+^, which has a λ_max_ around 475 nm.^32^ While this wavelength falls within the blue-green region, the complex appears red due to the absorption of its complementary color. Digital imaging devices, such as smartphones and cameras, operate similarly to the human eye in their perception of color. In analytical chemistry, RGB values extracted from digital images typically represent the complementary color of the absorbed light and are used as quantitative signals for analyte determination.^28^

Kajiya^33^ presents a chemistry lesson using anthocyanin-rich flowers and vegetables to explore the color wheel, with emphasis on the purple–green relationship. Students compare observed colors with absorbance spectra to understand the chemical basis of color perception, highlighting how the observed color corresponds to the complementary region of maximum absorbance.

## Acquiring Digital Images in Different Modes

Digital images can be captured under ambient lighting by placing cuvettes against a white background and photographing them with a smartphone, as illustrated in Figure 2. Alternatively, images may be obtained using a backlit background, such as a tablet screen (Figure 3), or with illuminated boxes equipped with light-emitting diodes (LEDs), as shown in Figure 4.^34^ Lighting conditions play a critical role in image quality; ambient light often introduces uneven illumination, which can compromise the consistency and accuracy of the data.

**Figure 2:**
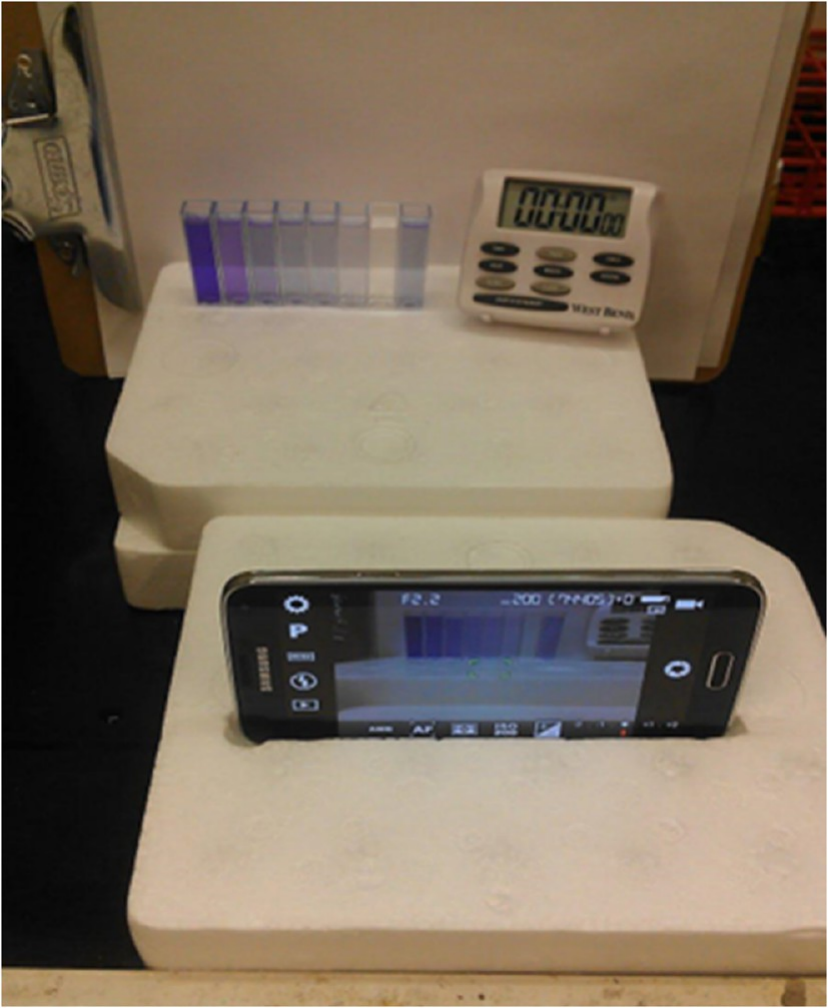
Experimental setup designated for crystal violet experiment (Figure reproduced from Knutson et al.^35^).

**Figure 3:**
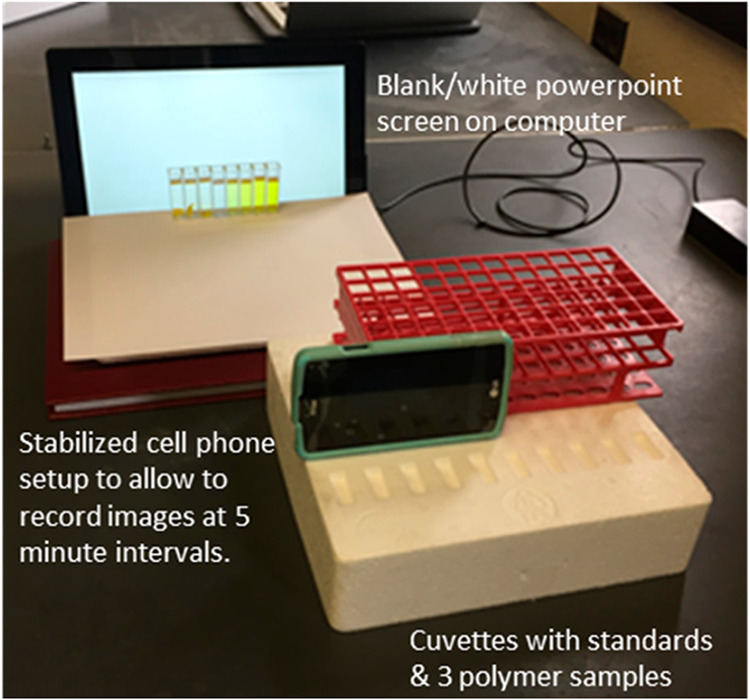
Sample setup for acquisition of images using a smartphone camera (reproduced from Knutson et al.^36^).

**Figure 4:**
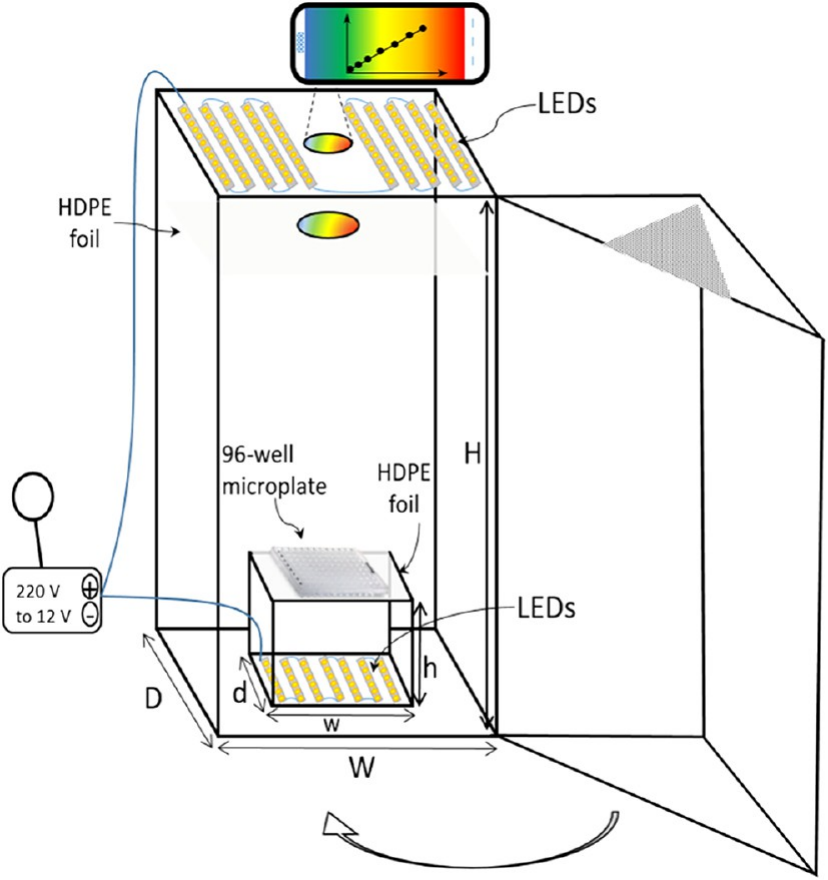
Diagram of the custom-designed apparatus used to capture digital images with a smartphone camera (overall dimensions: height = 21 cm, width = 15 cm, depth = 12 cm; inner compartment: *h* = 4 cm, *w* = 11 cm, *d* = 10 cm). (reproduced from Destanoğlu et al.^37^).

In some experiments, light passes through the sample, and the transmitted light is captured by a smartphone camera (Figure 5). In other setups, the sample is illuminated with LED light, and the resulting image of the illuminated sample is recorded (Figure 6). Figures 2, 3, and 4 demonstrate reflectance-mode imaging, in which the camera captures light reflected from the sample surface. Figure 5 illustrates transmittance mode, which closely resembles conventional UV–vis molecular spectroscopy. In contrast, Figure 6 depicts a fluorescence-like setup, where emitted light following LED excitation is recorded.

**Figure 5:**
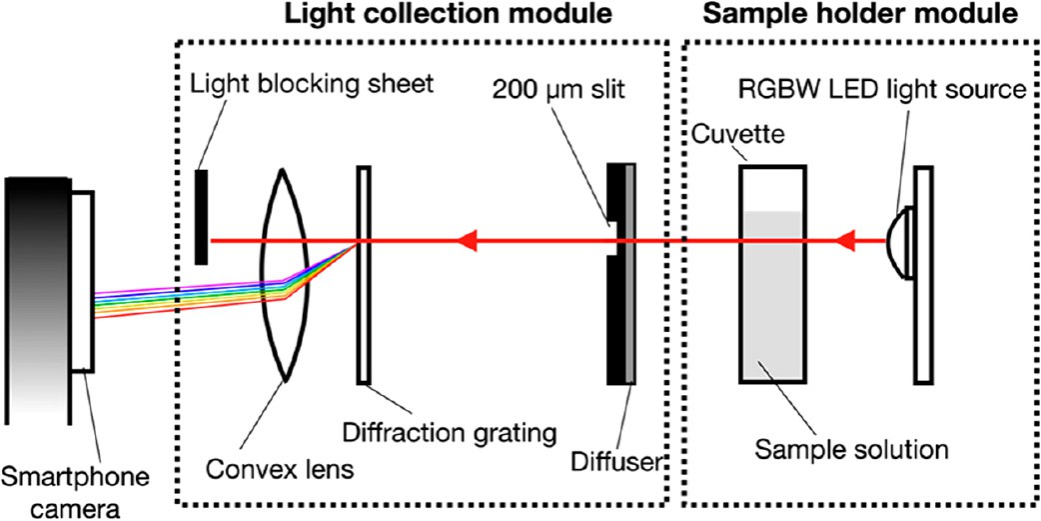
Acquiring digital images when light (LED) pass through the sample, and the transmitted light reach the smartphone camera. Reproduced from Jarujareet et al.^38^

**Figure 6:**
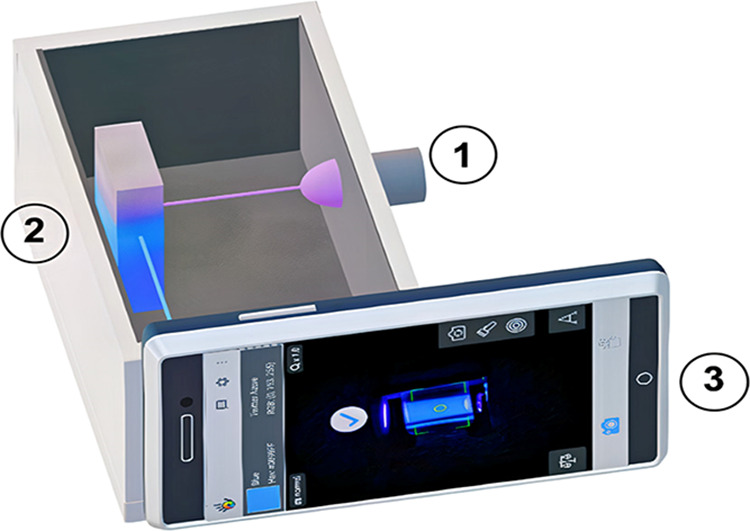
Diagram of the homemade device. Components: (1) excitation light source, (2) sample holder (polypropylene candy box), and (3) smartphone. The camera must be focused on the center of the sample holder. To insert and place the UV LED, make a hole as small as possible and paste it with tape; no spurious light should enter (Figure reproduced from Hamer et al.^39^).

## Factors That Affect Images Acquisition

The quality and reliability of analytical methods based on digital images can be influenced by several key factors, including ambient lighting conditions, device model (e.g., iPhone 7, 8, 14 Pro Max), brand (e.g., Samsung, Apple, Motorola), and the distance between the camera and the sample. Careful control and standardization of these parameters are essential to ensure reproducibility and accuracy in image-based measurements.

### Selection of the Channel

Kehoe and Penn^40^ established the foundational principles of quantitative analysis using digital images captured with smartphone technology. They demonstrated that the selected RGB color channel should correspond to the complementary color of the substance being analyzed, as illustrated in Figure 1. This complementary color is determined by the substance’s absorbance maximum (λ_max_). For example, the Fe­(SCN)^2+^ complex exhibits a λ_max_ around 475 nm, which falls within the blue region of the visible spectrum. Consequently, the complex appears red to the human eye, as it absorbs blue light and reflects red. In its quantification, the blue channel shows the greatest variation with concentration, compared to the red and green channels.^40,41^

### Focal Distance and Illumination

The focal distance—that is, the distance between the camera and the samples—strongly influences the results. To minimize variability, Kehoe and Lee Penn^40^ emphasized that both standards and samples should be captured within the same image (Figures 2 and 3). Uniform illumination is also essential for achieving reproducible results in digital image analysis. To address this, Quagliano and Marks^42^ developed an imaging setup in which a digital camera was fixed in position to maintain a constant focal distance (Figure 7). Their apparatus consisted of a black box illuminated by the screen of a tablet, ensuring consistent and homogeneous lighting conditions throughout image acquisition (Figure 7).

**Figure 7:**
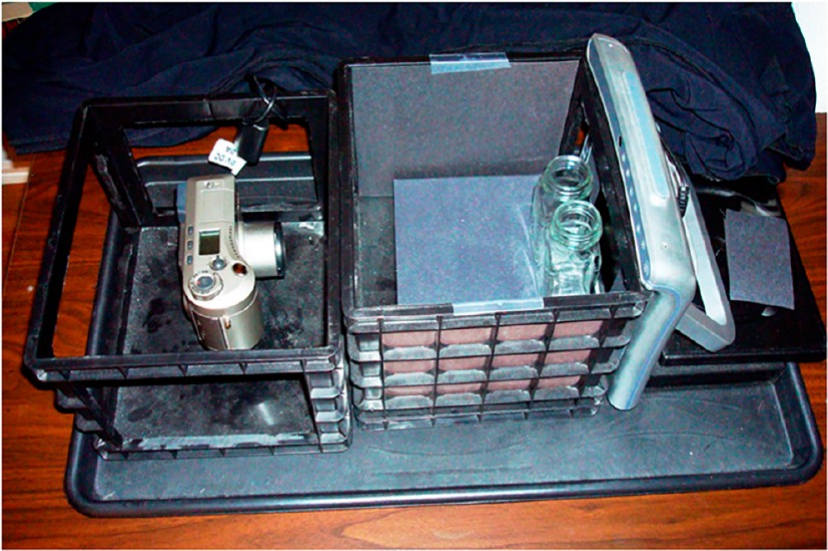
Photograph of the custom-built spectrometer; note the digital camera and the glass saltshaker cuvettes (Reproduced from Quagliano & Marks^42^).

Lateral flow assays (LFAs) are widely used for rapid, on-site molecular diagnostics. However, achieving high-precision results can be challenging and often requires a specialized optical setup to standardize the imaging environment. Park et al.^43^ introduced the Quick Light Normalization Exam (qLiNE), a method that enables conventional smartphones to function as reliable LFA readers. They demonstrated that when images are captured under ambient lighting using a smartphone, variations in the phone’s position can significantly impact signal intensity (Figure 8a). This issue, however, can be mitigated through signal normalization. As illustrated in Figure 8b, qLiNE effectively compensates for changes in smartphone angle and lighting conditions, resulting in consistent signal output.

**Figure 8:**
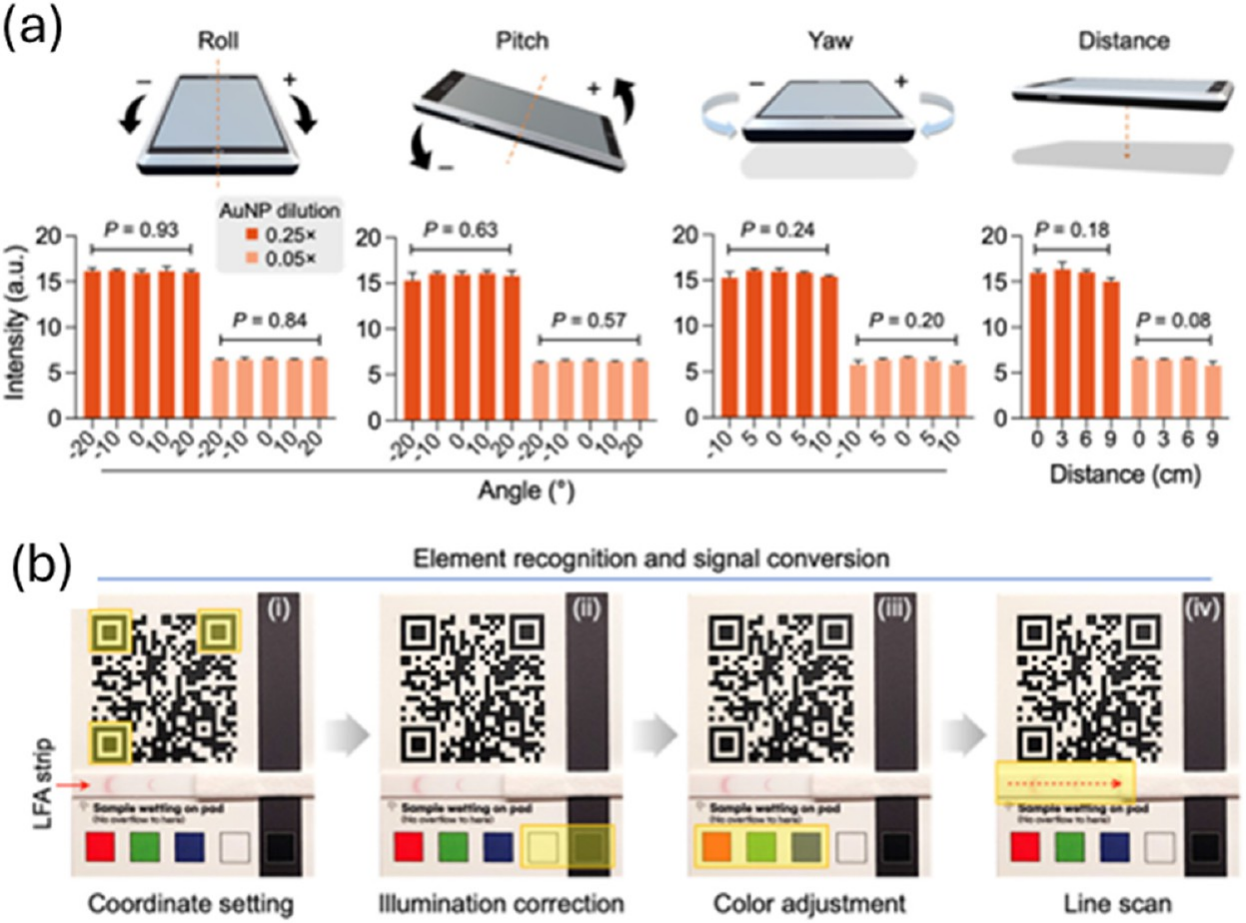
Image Processing with qLiNE. (A) The qLiNE workflow consists of several sequential steps: (i) the three corner markers within the QR code are detected to establish the image’s coordinate framework; (ii) illumination inconsistencies are corrected using predefined white and dark reference pads; (iii) color imbalances are normalized by calibrating the red, green, and blue channels, each using a designated color reference pad; (iv) finally, the color signal along the LFA test strip membrane is extracted. (D) LFA strips were photographed using a smartphone from varying angles and distances. Despite the variability in image acquisition conditions, the qLiNE app effectively corrected the images, yielding consistent signal intensities. For a fixed AuNP concentration, statistical analysis (one-way ANOVA) confirmed that the corrected intensity values did not differ significantly across the tested camera positions. Data are presented as mean ± standard deviation from duplicate samples. (reproduced from Park et al.^43^).

Maintaining a consistent focal distance is a critical factor for ensuring the reproducibility and accuracy of quantitative analyses based on digital images. To address this, Xiong et al.^44^ developed a simple yet effective apparatus that held the smartphone at a fixed distance from the samples. Although images were captured under ambient lighting conditions, the controlled spatial arrangement minimized variations in focal distance, thereby enhancing the precision and reliability of the analytical method.

At this point, it is important to emphasize that images acquired under controlled illumination conditions tend to yield more precise results than those captured under uncontrolled ambient lighting. Flatbed scanners, for instance, inherently provide consistent lighting, which enhances measurement reliability. Cebrián et al.^45^ demonstrated this effect by comparing RGB values obtained using a smartphone under ambient light versus controlled lighting or a flatbed scanner. They reported that the standard deviations of RGB values captured using a smartphone under ambient conditions were approximately twice as large as those obtained using a smartphone under controlled illumination and a flatbed scanner, indicating significantly reduced precision in uncontrolled environments.

### Devices from Different Brands

Unlike conventional analytical instruments—where equipment from different brands and models typically delivers comparable accuracy and precision—smartphones from different brands and models often introduce statistically significant variations in both measurement error and signal variability. Li et al.^46^ developed a smartphone-based system to monitor color changes of Meta-Cresol Purple in response to pH fluctuations, converting these colorimetric responses into quantitative pH values. Their study highlights the substantial influence that device selection can have on analytical outcomes. Smartphones differ widely in camera characteristics such as sensor sensitivity, white balance, and image processing algorithms—all of which can affect the accuracy and reproducibility of results. For example, Figure 9 presents a series of images of the same sample captured using different smartphones, revealing significant differences in color intensity and image quality. These variations underscore the critical role of device-specific camera characteristics in the accuracy and consistency of image-based analytical methods.

**Figure 9:**
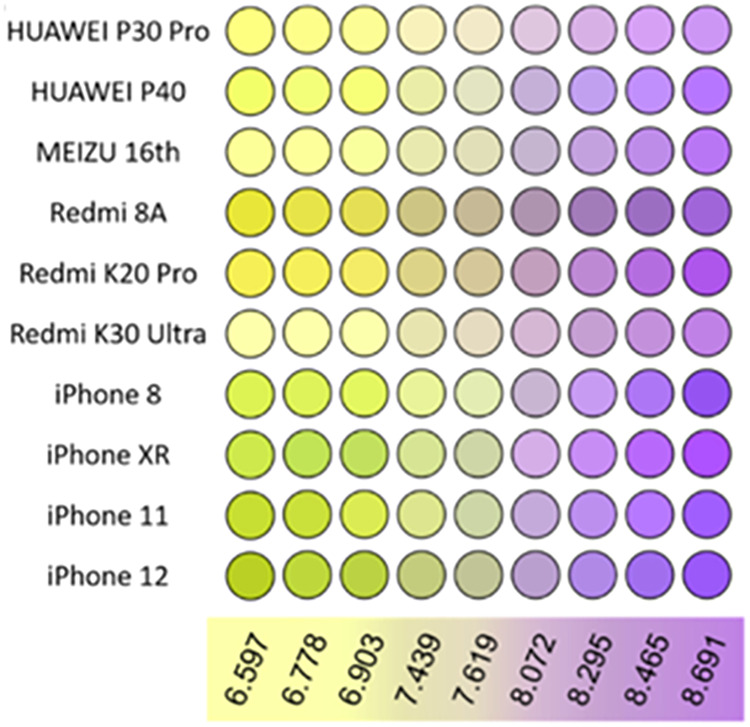
A series of images of the same sample, captured using different smartphones (reproduced from Li et al.^46^).

This variability is not limited to smartphones; it extends to other imaging devices such as scanners, digital cameras, and webcams. For example, measurements obtained using scanners from different brands have shown noticeable discrepancies. Similarly, Li et al.^46^ demonstrated that different smartphone models yielded varying degrees of measurement error (i.e., the difference between true and observed values) and signal standard deviations in the determination of seawater pH. These findings underscore the importance of rigorous calibration and standardization protocols when employing digital imaging in quantitative chemical analysis.

Cebrián et al.^45^ demonstrated that different devices produce varying responses. For example, in the quantification of H_2_O_2_ based on the oxidation of 3,3′,5,5′-tetramethylbenzidine (TMB), they found statistically significant differences among the Xiaomi Redmi 6A, Xiaomi Mi A2, and iPhone 8.

### Digital images File Format

Digital images can be saved in various file formats such as JPEG, PNG, and TIFF, and the choice of format can significantly impact analytical results. The commonly used RGB scale ranging from 0 to 255 corresponds to 8-bit color depth, which is standard in many digital systems. However, modern smartphones increasingly support higher bit depths, such as 10-bit (0-1023) and even 16-bit (0-65535), which offer finer gradation of color and potentially more accurate quantification in image-based analyses.

Nevertheless, not all file formats support higher bit depths. For instance, JPEG is limited to 8-bit color per channel, meaning that even if an image is captured with a 10- or 16-bit capable camera, saving it as a JPEG compresses it down to 8-bit, resulting in loss of detail and dynamic range. In contrast, formats such as TIFF, DNG, and Apple ProRAW can preserve higher bit-depth data, allowing for more precise color analysis.

For example, the iPhone 12 is equipped with a 10-bit imaging system, but to retain this advantage, images must be saved in formats that support high bit depth (e.g., TIFF, DNG, or Apple ProRAW). If saved as JPEG, the data are effectively reduced to 8-bit, negating the hardware’s higher bit-depth capability.

However, when using a camera or device that only supports 8-bit output, the choice of file format (e.g., JPEG vs TIFF) does not typically result in significant differences in analytical performance. For example, Figueiras et al.^47^ reported equivalent sensitivity, limits of detection (LOD), and quantification (LOQ) for nitrite analysis using 8-bit images saved in both TIFF and JPEG formats.

Digital cameras and smartphones typically contain twice as many green pixels as red or blue in their image sensors, making them inherently more sensitive to green light. To generate full-color images, devices apply a process called Bayer interpolation, which estimates missing color values and adjusts the intensity of RGB channels to create a visually balanced image. While this is ideal for aesthetic purposes, it introduces significant alterations to the raw sensor data—an issue that severely compromises accuracy when conducting quantitative analyses based on RGB values.

Furthermore, a growing concern is the widespread use of artificial intelligence (AI) in smartphone image processing. AI-driven enhancements such as automatic color correction, contrast adjustment, and sharpening may further distort the true color intensities recorded by the sensor. These modifications, while producing more visually appealing images, undermine the reliability of the data for scientific measurements.

To ensure accurate and reproducible results, it is essential to bypass these processing steps whenever possible. Capturing images in RAW format preserves the original sensor data without interpolation or AI-based enhancements, making it the preferred choice for quantitative digital image analysis.

## Economic and Practical Viability

Quantitative analyses conducted in transmittance mode, as shown in Figures 2 and 3, incurred no additional cost—provided that students or researchers already owned smartphones.^48^ Although conventional photometers are relatively affordable, their prices are often only marginally higher than those of some smartphones or scanners. For instance, in Brazil, a commercial portable photometer can be less expensive than an iPhone. Consequently, from a purely economic standpoint, using a smartphone in place of a photometer may not be cost-effective, especially when factoring in the additional time required to process images into analytical signals. Nonetheless, the educational value is significant: allowing students to use their own smartphones to quantify unknown samples fosters engagement, autonomy, and experiential learning.

Microplate readers—widely used in high-throughput analyses—commonly utilize 96-well plates, though configurations ranging from 6 to 1536 wells exist, accommodating volumes from 5 to 200 μL.^49^ These instruments offer notable advantages over traditional cuvette-based methods (which typically use 1–5 mL),^47^ including reduced reagent consumption and the ability to analyze large numbers of samples quickly. However, their high cost can be prohibitive. In this context, replacing commercial plate readers with smartphone-based digital imaging offers a substantial economic advantage, delivering comparable functionality with significantly lower financial investment.

## Advantages and Disadvantages

### Disadvantages

Spectroscopy is a fundamental and widely used tool in chemistry.^50^ Among the various spectroscopic techniques, UV–visible (UV–vis) spectrophotometry stands out as one of the most employed methods, serving as a cornerstone in both educational and research settings.^51^ Despite its broad application, undergraduate instruction often introduces UV–vis spectroscopy through idealized models, which may overlook the practical complexities and limitations encountered in real-world analyses.^51^

When using commercial UV–vis spectrophotometers, the linear range of absorbance is typically limited, with reliable measurements falling between approximately 0.1 and 1.2 absorbance units.^50,51^ A similar limitation applies to digital image-based measurements, although their linear range is often slightly narrower. This is primarily because commercial UV–vis spectrophotometers utilize narrow spectral bands, whereas digital cameras rely on broad bandpass filters, which reduces spectral resolution and, consequently, narrows the quantitative range. Moreover, the signal intensities captured through digital imaging—whether in transmittance, reflectance, or fluorescence modes—are typically lower than those obtained using conventional laboratory-grade instruments.^47,52–54^ For example, Figure 10 shows the small range obtained in a fluorescence experiment.

**Figure 10:**
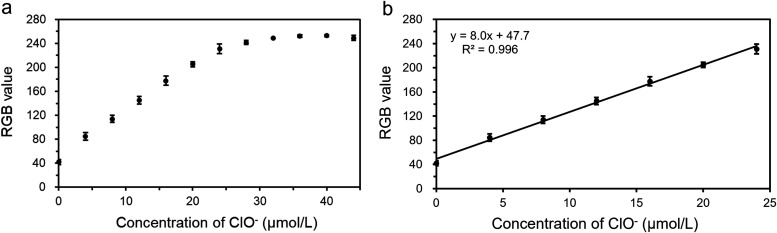
(a) Calibration curve illustrating the relationship between RGB values and ClO^–^ concentration in the range of 0–44 μmol/L. (b) Linear fit of RGB values versus ClO^–^ concentration within the 0–24 μmol/L range, where the response remains linear. (reproduced from Lu et al.^52^).

The sensitivity of methods based on digital images is typically about three times lower than that of conventional analytical instruments. Therefore, the main disadvantages of digital imaging compared to commercial equipment are its narrower linear range and lower sensitivity.

### Advantages

Affordability is one of the most compelling advantages of using flatbed scanners or smartphones as alternatives to 96-well plate readers, which are typically expensive and often inaccessible in resource-limited settings. This economic benefit becomes especially valuable in reflectance mode applications (as illustrated in Figures 2 and 3), where reflected light, rather than transmitted light, is measured. Reflectance-based imaging enables the analysis of solid samples and turbid liquids containing suspended particles—sample types that are often incompatible with conventional UV–vis spectrophotometry due to light scattering.

Another advantage of using smartphones is their built-in connectivity features, such as Internet access and GPS, which enable geolocated chemical analysis. This allows users to determine the concentration of a contaminant at a specific location and instantly upload the data online, facilitating the creation of real-time, global contamination maps. For example, Pol et al.^55^ demonstrated this approach by mapping nitrite concentrations across a defined geographic area.

For example, milk, which contains suspended fat globules and proteins, cannot be reliably analyzed using UV–vis spectroscopy because light scattering distorts absorbance measurements. In contrast, reflectance mode is largely unaffected by such scattering, allowing for direct and accurate analysis. Malschitzky et al.^56^ exemplified this approach by quantifying hydrogen peroxide (H_2_O_2_) in cow’s milk using digital images acquired with a flatbed scanner.

Similarly, Prussian blue, a colloidal solid formed in solution, poses challenges for UV–vis analysis due to its strong light-scattering properties. However, this limitation is overcome in reflectance mode. Filgueiras et al.^47^ employed the Prussian blue reaction to determine iron content in dietary supplements using digital image analysis, demonstrating the versatility and analytical utility of reflectance-based methods when combined with low-cost imaging technologies.

## Converting RGB Values in Analytical Signals

Among the various available color spaces, the RGB color space is the most frequently employed in the cited articles. Typically, digital images are captured and subsequently processed on a computer to extract RGB values using software such as MATLAB,^57^ the R project,^58^ and ImageJ.^47,53,54,59–61^ Notably, ImageJ, a freeware, has emerged as the most widely used software for this purpose.^62^ In some instances, RGB values are directly measured using smartphone apps, such as MIT App,^63^ Color Grab,^59,64^ ColorAssist,^64^ and Color Picker.^65^ Digital images acquired via cameras, smartphones, webcams, tablets, and flatbed scanners find numerous applications in chemistry education. Their initial use was to replace photometers in laboratory experiments for quantitative analysis. In most of the studies, RGB values were converted into an analytical signal using eq 1, where I_0_ represents the RGB values of the blank, and I denotes the RGB values of samples and standards. The equation used is as follows:

1 $$S=-\log\left(I/I_{o}\right)$$

In reflectance and fluorescence modes, *I* represents the light reflected by the sample, while *I*_0_ corresponds to the light reflected by a blank reference. Similarly, in transmittance (absorbance) mode, *I* is the light transmitted through the sample, and *I*_0_ is the light transmitted through a blank reference.

Equation 1 [Disp-formula eq1] was the most commonly used method for converting RGB values into an analytical signal (Figure 11). This approach is exemplified in Figure 11a, where only the green channel exhibits significant variation. This behavior is attributed to the fact that green is the complementary color of the pink compound formed in response to the analyte. In this example, nitrite was quantified using the Griess assay, which produces a pink-colored azo compound. As a result, the green channel showed the most pronounced changes with increasing nitrite concentration.^47^

**Figure 11:**
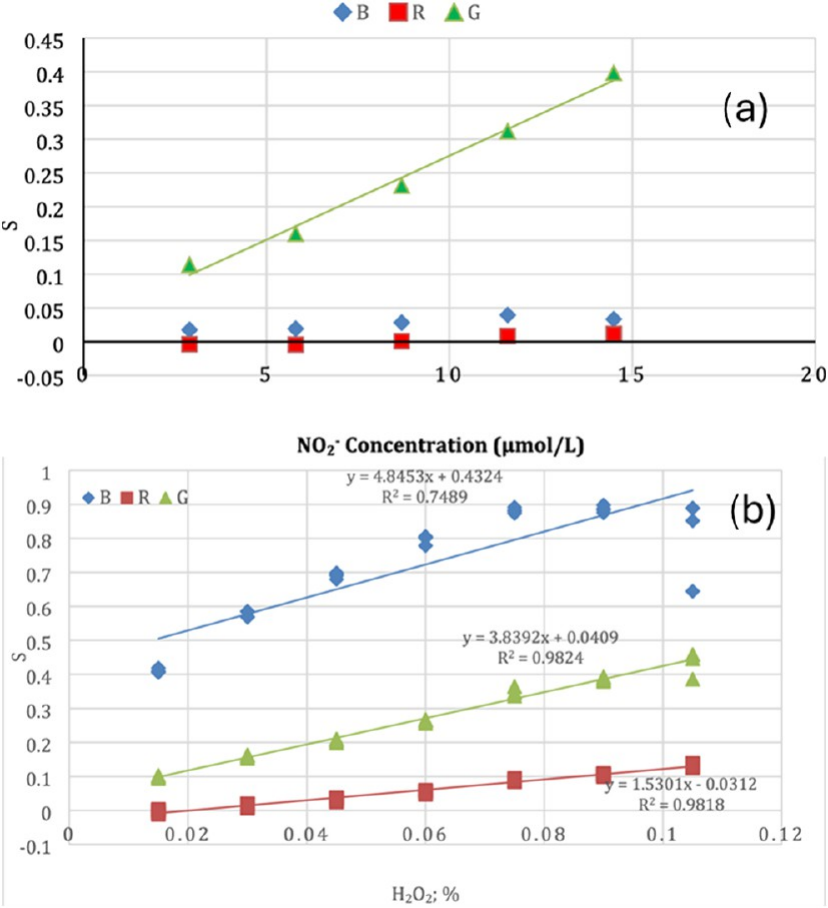
External calibration plots for (a) nitrite quantification using the Griess assay (reproduced from Filgueiras et al.^47^) and (b) hydrogen peroxide quantification based on the reduction of iodide (I^–^) to iodine (I_2_) (reproduced from Malschitzky et al.^56^).

In contrast, Figure 11b presents the quantification of hydrogen peroxide (H_2_O_2_) in milk via the reduction of iodide (I^–^) to iodine (I_2_), forming a yellow-colored solution. In this case, all three RGB channels varied with analyte concentration. Although the blue channel (the complementary color of yellow) exhibited the largest overall change, the green channel was selected for quantification because it provided better linearity across the studied concentration range.^56^

Several alternative eqs can be employed to convert RGB values into analytical signals. In the quantitative analysis of ethanol in distilled beverages, Filgueiras et al.^66^ determined ethanol content by exploiting its ability to suppress the ionization of phenolphthalein. As ethanol concentration increased, the characteristic pink color diminished accordingly.

Using eqs 1 and 2, the green channel—complementary to the pink coloration—exhibited the most significant variations relative to ethanol concentration. Equation 3, in contrast, incorporated normalization of RGB channels via eq 4. Upon normalization, the red channel displayed the greatest sensitivity to changes in ethanol content. Although eqs 1–3 yielded statistically equivalent results, eq 3 occasionally produced broader interquartile ranges, suggesting slightly higher variability under certain experimental conditions, where q. One was recommended due to its simplicity.

2 $$S=R-R_{0}$$

3 $$S=-\log\left(\frac{R^{\mathrm{nor}}}{R_{0}^{\mathrm{nor}}}\right)$$

4 $$R^{\mathrm{nor}}=\frac{R}{\sqrt{R^{2}+G^{2}+B^{2}}}$$

Normalization of RGB values can shift the channel that exhibits the greatest variation as a function of analyte concentration to the one that more directly reflects the perceived color of the solution. For example, in the determination of the equilibrium constant for the reaction between iron­(II) and phenanthroline, the product is an orange-red complex. Since the authors used normalized RGB values, the red channel—corresponding to the color of the complex—showed greater variation with complex concentration than the blue and green channels, even though blue is the complementary color of red.^67^

In some cases, normalization of RGB values can help reduce the standard deviation of the data.^53^ For example, in the quantification of casein using the Bradford assay (Figure 12a), the external calibration constructed with unnormalized RGB values exhibited greater variability. When RGB values were normalized, the standard deviation decreased, leading to improved precision of the analytical method (Figure 12b).

**Figure 12:**
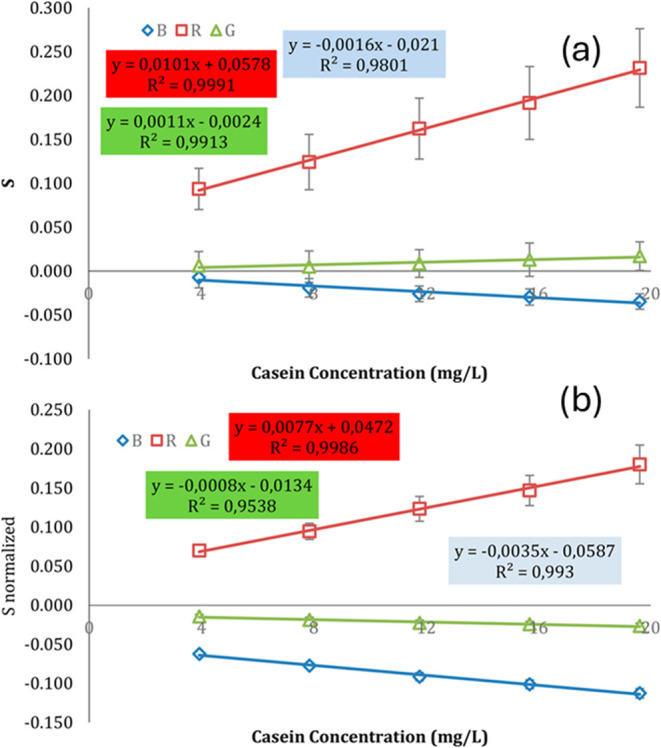
External calibration curves for casein quantification using the Bradford assay. (a) Calibration based on unnormalized RGB channel values; (b) calibration using normalized RGB channel values (reproduced from Filgueiras et al.^53^)

Lopez-Molinero and Jimenez-Lamana^68^ quantified the concentration of H_2_O_2_ in commercial products using TiO_2_ in an acidic medium, where increasing H_2_O_2_ levels intensified the yellow coloration. Among the RGB channels, the blue channel—complementary to the observed color—exhibited the most pronounced variations as a function of H_2_O_2_ concentration. The authors applied a series of equations (e.g., eqs 5 and 6), using only the blue channel values to establish the relationship between the analytical signal and H_2_O_2_ concentration. This approach yielded the narrowest linear range and the highest measurement precision.

5 S=(R+G+B)

6 S=B/(R+G+B)

Several studies have employed chemometric techniques,^69^ such as K-Nearest Neighbors (KNN) and Partial Least Squares (PLS),^70^ for quantitative analysis. Both KNN and PLS utilize training data sets to calibrate models capable of predicting analyte concentrations. For instance, the PhotoMetrix app implemented these approaches to quantify analytes,^71,72^ demonstrating their effectiveness in diverse applications including Chemical Oxygen Demand (COD) measurement,^73^ copper quantification in alcoholic beverages,^74^ and manganese detection in water samples.^75^

## Flatbed Scanners

Most of the papers cited in this review employed reflectance mode, with digital images captured using smartphones. Indeed, the majority of studies involving digital imaging in chemical analysis have utilized smartphones due to their accessibility and convenience. However, flatbed scanners represent a compelling alternative, particularly for imaging 96-well plates. Scanners offer a fixed focal distance and uniform illumination, making them especially attractive for quantitative analysis. Moreover, they enable automatic extraction of RGB values, streamlining the image processing workflow.

Soldat et al.^76^ introduced automated extraction of RGB values for the first time using a plugin in ImageJ, enabling the simultaneous extraction of RGB values from all wells in a 96-well plate. Subsequently, a plugin with a visual interface for the same purpose was developed under the name “ReadPlate”.^77^ This software has been extensively utilized for the automated extraction of RGB values from images of 96-well plates in various research works.^47,53,54,56,60,61,66,78–81^

## Quantitative Analysis

This section discusses some of the analytes frequently examined in laboratory experiments utilizing digital images. Most of the laboratory experiments described in the literature focused on quantitative analysis, where RGB values were used instead of absorbance. There were few reports of other techniques, such as flame emission.

### Food Dyes

The first manuscript published in the Journal of Chemical Education (JCE) was the work by Kohl et al.^2^ In their study, they photographed yellow food dye solutions with known relative concentrations placed in cuvettes against a diffuse fluorescent white lightbox. They created a calibration plot by extracting RGB values using ImageJ. Kohl et al.^2^ emphasized the importance of using a uniform background to reduce ambient light interference and the need to capture all samples in a single image to ensure consistent camera-to-sample distances. Their imaging setup employed 96-well plates, with the scanner effectively shielded from external light sources to enhance reproducibility. Since the food dye displayed a yellow hue, the blue channel—its complementary color—exhibited the most significant variation in response to changes in dye concentration.

Subsequently, Quagliano & Marks^82^ detailed a series of experiments for dye quantification, where solutions were photographed from a fixed distance against a digital photo frame as the background (Figure 7). The laboratory experiment configuration for quantitative analysis using digital images as a detector was introduced by Kehoe & Penn.^40^ In this experiment, blue food dye concentrations were quantified by placing cuvettes containing standards and unknowns against a white background. A digital image was then captured using a smartphone, and RGB values were extracted using ImageJ. A variation of this experiment was adapted for at-home use during the COVID-19 pandemic.^48^ Food dyes were also quantified using smartphones in a microextraction laboratory experiment.^83^

Barbosa et al.^84^ developed a smartphone-based method for determining the concentration of Sunset Yellow dye using a simple paper device. Designed for use during the COVID-19 pandemic, this at-home experiment involved capturing images of the paper strip under ambient lighting using a smartphone camera. The RGB values were extracted from the images using ImageJ software. Among the channels, the blue channel exhibited the most pronounced variation with respect to dye concentration. The method demonstrated a linear detection range of 10–80 mg/L.

Ambruso & Riley^85^ determined the concentration of Allura Red in maraschino cherry juice. Both standards and samples were placed against a white background, typically a white sheet of paper. The entire process, including RGB value extraction, constructing the standard curve, and quantifying the unknown, was facilitated by smartphone apps such as “Color Name” (for iPhone) and “Colorimeter” (for Android). The concentration of Allura Red was quantified within the range of 10–40 mg/L.

Grasse et al.^86^ addressed the influence of ambient light by designing an innovative 3D-printed apparatus (Figure 13). This setup effectively minimized external light interference and enabled the acquisition of absorbance versus wavelength plots, allowing for the precise quantification of dye concentrations in powdered drink samples. The apparatus exemplifies a transmittance-based approach, in which the light passing through the sample is captured by the camera for analysis.

**Figure 13:**
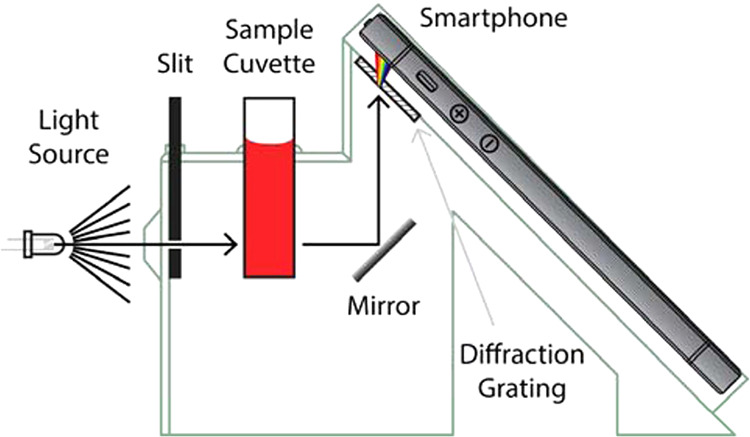
Schematic of the SpecPhone. A stable light source enters the SpecPhone apparatus and is spatially filtered by a removable slit that controls the geometry and intensity of light projected through the sample. The light transmitted through the sample is reflected off a mirror and through the diffraction grating placed at a 45° angle. This grating disperses the light into its color spectrum and into the smartphone camera, which can be viewed on the screen and saved as a photo. (Reproduced from Grasse et al.^86^).

Hosker^63^ introduced a shoebox spectrophotometer, which comprises a shoebox, an LED light source, and a DVD acting as a diffraction grating (as illustrated in Figure 14). The analytical signal was determined using a smartphone app developed for the shoebox. This app measured the intensity of light and translated it into absorbance values. Using this device, the concentrations of Brilliant Blue and p-nitroaniline were quantified within the linear range of 0.5–2.5 mg/mL and 0.1–0.5 mM, respectively.

**Figure 14:**
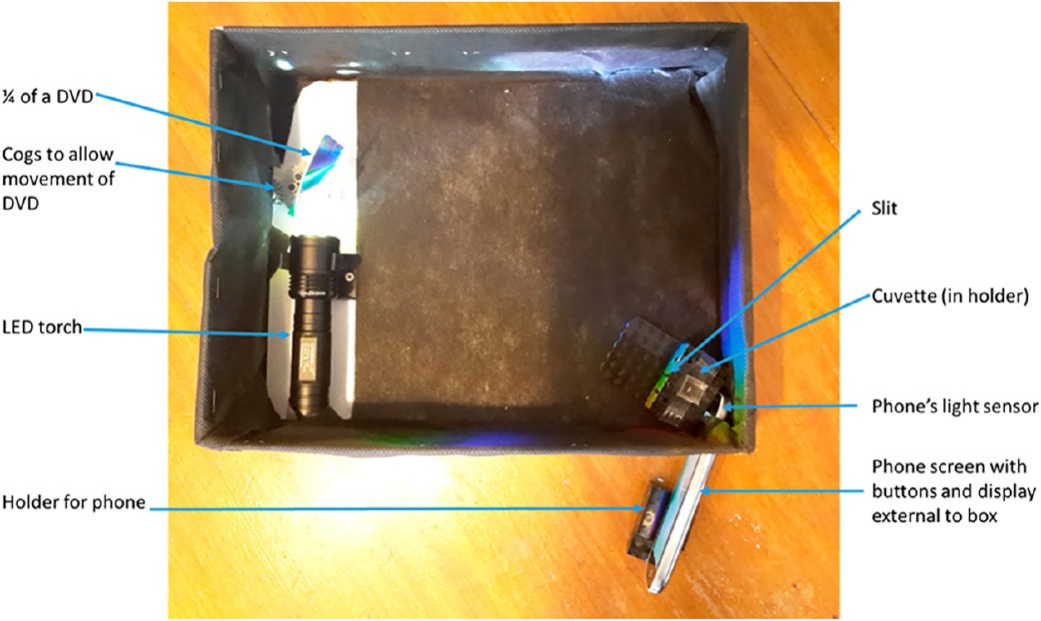
Shoebox spectrophotometer (Reproduced from Hosker^63^).

In a manner like that of a shoebox spectrophotometer, a new shoebox spectrophotometer was developed to obtain the emission spectra of Na^+^ and Cu^2+^ and the absorption spectra of permanganate.^87^

### Iron­(III)

Iron­(III) is a common analyte, and Armenta et al.^88^ quantified it in water samples using paper devices (Figure 15) based on its reaction with thiocyanate. Photographs of the paper devices were taken under ambient light conditions using smartphones. The gray values were then extracted using various software applications, including ImageJ, Hypocam (version 2.2.1), MATLAB, and GIMP 2. The method exhibited a linear range of 10–90 mg/L.

**Figure 15:**
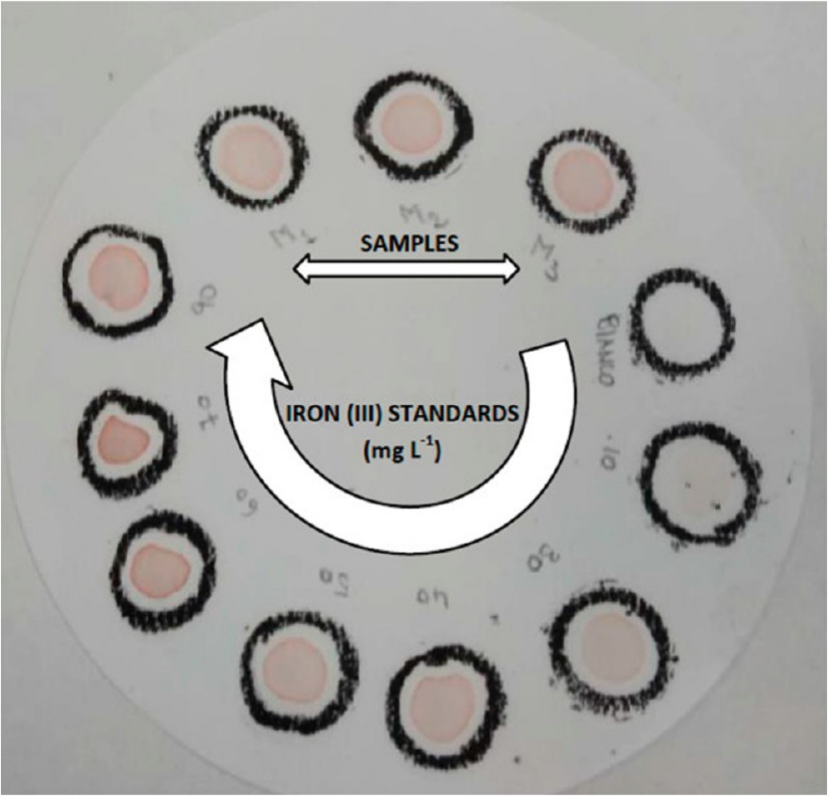
Paper devices used to quantify Fe^3+^ concentration in water sample (Figure reproduced from Armenta et al.^88^).

Zhang et al.^89^ developed a smartphone application that automatically constructs an external calibration curve and determines the concentration of unknown samples. The app was validated through the quantification of iron­(III) using its colorimetric reaction with KSCN. The experiment was performed in reflectance mode, as shown in Figure 3, with a notebook screen serving as the background light source and the smartphone fixed at a constant distance from the cuvette.

Iron­(III) was quantified using anthocyanins extracted from *Ruellia tuberosa* L.^90^ The reaction between iron and anthocyanin resulted in a pink color change. Samples and standards were placed on a porcelain plate, which was then positioned in an illuminated black box, and photos were acquired using smartphones. Blue values were extracted using ImageJ, and these values were used to quantify the iron content. The method exhibited a linear range of 0.1 to 2.0 mg/L.

Iron­(III) was also analyzed using curcumin paper, where it formed a complex with curcumin on a paper substrate, causing a color change from yellow to red-orange. RGB values were obtained directly from smartphones using the app “Color Grab” and indirectly using ImageJ. Red and green values provided higher sensitivity and *R* ^2^ values close to 1. In this method, students were encouraged to choose the most suitable color channel. The linear range for this approach was 0.05 to 0.25 μmol/L.^59^

In another laboratory experiment, Iron­(III) was quantified using three different chromogenic derivatizing reagents (thiocyanate, 1,10 phenanthroline, and FerroZine).^43^ Fe^3+^ samples and standards were placed in paper filter disks, followed by the addition of the respective chromogenic reagents. Phenanthroline and FerroZine specifically react with Iron­(II), necessitating the prior reduction of Iron­(III) to Iron­(II) using hydroxylamine hydrochloride. Colorimetric measurements were directly obtained using color-detecting apps such as “Color Assist” and “Color Grab” (for Android). Students had the flexibility to select the optimal color channel, with green commonly chosen for FerroZine, blue for thiocyanate, and o-phenanthroline. This experiment enabled students to explore figures of merit and observe that the FerroZine method exhibited higher sensitivity than the other two methods.^91^

Filgueiras et al.^60^ demonstrated the quantification of Iron­(III) using several chromogenic reagents in laboratory experiments. Salicylic acid, gallic acid, K_4_[Fe­(CN)_6_], and thiocyanate assays were used as chromogenic reagents. These reactions produced blue, red, blue, and red products, respectively. The optimal channels for quantitative analysis were R, B, R, and B, respectively. Linear ranges for these assays were 4–20, 2–9, 1–5, and 1–5 mg L^–1^, respectively. Samples and standards were placed in a 96-well plate, and images were obtained using a flatbed scanner. The study also compared external and standard addition calibration techniques.^60^ Students were able to observe that λ_max_ was related to the complementary color and could compare the different limit ranges obtained for various chromogenic reagents.

### Fluoride

Students quantified fluoride in black tea, green tea, toothpaste, and mouthwash through its reaction with (E)-2-(1-(6-nitro-2-oxo-2H-chromen-3-yl)­ethylidene)-*N*-phenylhydrazine-1-carbothioamide (CT).^92^ This compound was then used to react with the samples, resulting in the formation of a pink color in the presence of fluoride. The concentration of fluoride was determined by calculating R/G ratios. R and G values were determined using the app Color Picker, with tubes containing standards and samples placed in a white box. R and G values were directly obtained using a smartphone, but data processing was performed on a computer. This method demonstrated a linear range of 1–10 ppm. In addition, students synthesized the CT chromogenic reagent and characterized it using physical methods. They also learned about Job’s method and the selectivity of the synthesized compound for fluoride, as it did not present any color change for ions such as NO_3_^–^, H_2_PO_4_^–^, Cl^–^, acetate, Br^–^, SO_4_^2–^, I^–^, CN^–^, and oxalate.

### Proteins

The most commonly used software for extracting RGB values from digital images was ImageJ. Among the various applications for this purpose, ColorX was specifically introduced for the quantification of MnO_4_^–^, Co^2+^, Cu^2+^, Ni^2+^, and proteins using the Lowry assay.^93^ Protein concentrations were quantified in the range of 1–10 mg L^–1^.

Proteins were also quantified using the Bradford and biuret assays.^94^ The methodology followed was like that previously presented by Kehoe & Penn.^40^ The linear range for the biuret assay was 0.5 to 3 g L^–1^, and for the Bradford assay, it was 0.5 to 3 mg L^–1^. Furthermore, proteins were quantified using images captured in 96-well plates. The total protein content in whey protein samples was determined using the Lowry assay, within a range of 0.1 to 1.2 mg L^–1^.^80^ Images of 96-well-plates were acquired using smartphones under ambient light conditions. As the Lowry assay produces a blue color, red values were employed to quantify the total protein content.

The total protein content in beer, milk, and whey protein was quantified using the Bradford assay.^53^ Digital images were acquired using a flatbed scanner, and the method exhibited a linear range of 4 to 20 mg L^–1^. The detection limits and quantification limits (LOD and LOQ) were found to be 1.52 and 4.62, respectively. Barton et al. determined protein concentrations using the bicinchoninic acid assay, utilizing images from 24-well plates captured with a smartphone.^95^ Given that the bicinchoninic acid assay yields a purple color, the G channel was utilized for quantification. The linear range for this method was 0.1 to 1 mg/L.

### Starch in Banana

Doughan & Shahmuradyan quantified the amount of starch in bananas through a reaction involving starch and iodine.^96^ This experiment was devised as an at-home activity during the COVID-19 pandemic. Standards and samples were photographed using a smartphone under ambient lighting conditions against a white background. The photos were subsequently processed on a computer using ImageJ for quantitative analysis. The red values were used for quantification, and the method exhibited a linear range of 5–35 mg L^–1^.

### Cu­(II)

Kuntzleman & Jacobson^97^ detailed a method for quantifying copper using a simple setup consisting of a shoebox. In this setup, a cuvette was placed within the shoebox against a red background, and the *R*-values were directly determined using a smartphone app (as depicted in Figure 16).

**Figure 16:**
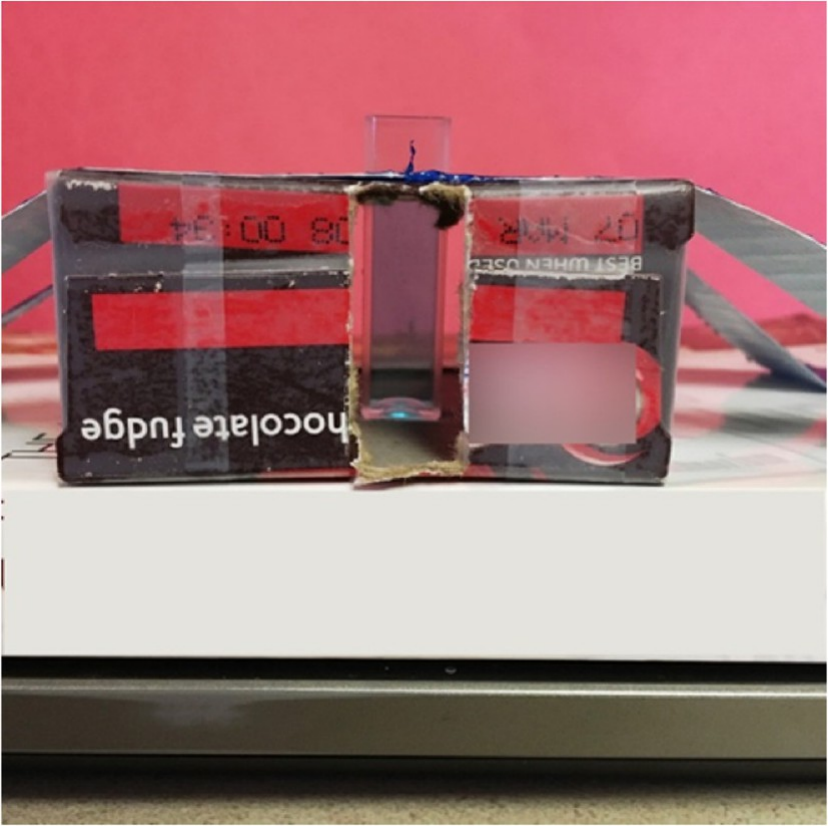
View of blank (left) and sample of 0.50 M CuSO4 (right) through the RGB analyzer on a smart phone. The application records the average R, G, and B values of the pixels within the circle (see lower right-hand corner of each image). Given the R values for the blank (190) and the sample of CuSO4. (Figure reproduced from Kuntzleman & Jacobson^97^).

Montangero^64^ determined copper concentrations in volumetric flasks directly using an app by measuring Hue values. The copper concentration was determined using just three standard solutions (0.2, 0.4, and 0.6 mol L^–1^). Subsequently, Zhu and Ling^98^ demonstrated that the Hue values remained constant and did not change with variations in copper concentration.

### Pharmaceuticals

Ciprofloxacin concentrations in pharmaceutical pills were determined using paper microfluidics.^99^ Ciprofloxacin’s 1,3-dicarbonyl group forms a complex with FeCl_2_, resulting in a vivid orange coloration. Digital images of the paper microfluidic devices were captured under ambient lighting conditions using smartphones. The blue channel intensities were then extracted using ImageJ software to quantify ciprofloxacin concentrations. The quantitative analysis followed the method described by Park et al.^43^ (Figure 8), which incorporates features in the paper microfluidic devices to correct for nonuniform illumination and variations in smartphone positioning and distance relative to the devices (Figure 17). Despite these corrections, students experienced low coefficients of determination, indicating poor linearity in their calibration results.

**Figure 17:**
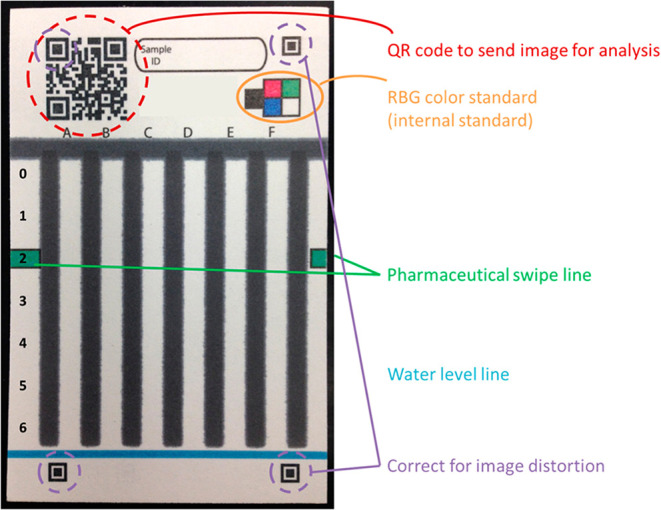
Paper analytical devices were fabricated by printing hydrophobic barriers using a Xerox ColorQube solid ink printer, followed by heat treatment in an oven to fix the patterns. Specific reagents were then applied at designated locations along the lanes, corresponding to the targeted colorimetric assays (reproduced from Bliese et al.^99^).

The concentrations of acetylsalicylic acid in pharmaceuticals were quantified through hydrolysis in alkaline media, followed by a reaction with iron­(III) to form a purple complex.^54,100^ The standards and solutions were placed in a 96-well plate, and images were obtained using both a flatbed scanner and a smartphone. In both studies,^54,100^ the green values (complementary to purple) were used to quantify the acetylsalicylic acid concentration.

The concentration of gold nanoparticles in MesoGold, a dietary supplement, was determined within a range of 5–50 ppm.^101^ Standards and samples were placed in cuvettes against a white background (like Figure 2). Subsequently, a digital image was obtained under ambient light using a smartphone. The quantitative analysis was performed using the green values extracted from the digital images, as the color of MesoGold is pink, and it changes with the concentration of particles in the solution. The concentration of gold nanoparticles cannot be determined using UV–vis due to the light-scattering properties of the nanoparticles, but it can be accurately determined using digital images.

Acetaminophen reduces ferricyanide to ferrocyanide, converting to the highly colored Prussian Blue through the addition of iron­(III). This approach was used to quantify acetaminophen in pharmaceuticals. The red values were extracted directly from test tubes under ambient light using smartphones.^102^

In a thin-layer chromatography (TLC) laboratory experiment, disulfiram was quantified in Anticol tablets. The tablets were dissolved, and the disulfiram was separated from the excipients using TLC. Quantitative analysis was performed using the multiple standard addition method. An image of the TLC plate was then obtained using a flatbed scanner, and the size of the standard and standard-added samples was determined using ImageJ.^103^

### Starch

The starch concentration in potatoes was determined using the starch–iodine reaction. Both samples and standards were placed in a 96-well plate, and an image of the plate was captured using a flatbed scanner. RGB values were then extracted using specialized software designed for quantitative analysis based on digital images.^104^

### NO_3_^–^

Bogucki et al.^105^ optimized the SpecPhone (as shown in Figure 13) for a dual-beam optical geometry. This setup was employed to quantify nitrate concentrations within the range of 1–10 mg L^–1^.

### NO_2_^–^

Nitrite was quantified in food samples, including bacon, sausage, and spinach, using the Griess assay.^47^ In this procedure, 5 g of food were boiled with a borax solution, and fats were removed using potassium ferrocyanide (K_4_[Fe­(CN)_6_]) and zinc sulfate (ZnSO_4_). The resulting samples and standards were placed in a 96-well plate, and images were acquired using a flatbed scanner. Nitrite concentrations were quantified within the range of 2.9–14.5 μmol L^–1^. In the Griess assay, the reaction produces a pink diazonium compound with a maximum absorbance (λ_max_) at 540 nm. Accordingly, the green color channel showed the most pronounced changes in response to varying nitrite concentrations. This experiment provided students with a hands-on opportunity to work with solid food samples, including nitrite extraction, sample preparation, and quantitative analysis.

### Biodiesel

The percentage of biodiesel in diesel-biodiesel blends was quantified by tracking the bathochromic shift of Nile Blue, which produces a color change proportional to biodiesel concentration.^106^ Key variables—including diluent (ethanol) ratio, Nile Blue concentration, blend composition, pH, and temperature—were systematically adjusted due to their influence on the bathochromic shift magnitude. Samples and standards were imaged individually within a controlled, LED-illuminated chamber to maintain consistent lighting conditions. Quantification was based on red channel intensity values extracted using ImageJ software. This approach exhibited a linear response for biodiesel content between 0 and 40%.

### Folin–Ciocalteu Assay

The Folin-Ciocalteu assay, a widely used method for assessing total phenolic content in foods, was applied to quantify the phenolic content in beer samples. Both samples and standards were loaded into a 96-well plate, which was then imaged using a flatbed scanner.^61^ The Folin-Ciocalteu reagent, originally yellow, reacts with phenolic compounds to produce a blue color. This color change resulted in significant variations in the red channel intensity of the images. Red channel values from all wells were automatically extracted using the ReadPlate plugin in ImageJ. Using gallic acid as the calibration standard, the method demonstrated a linear range of 2–10 mg L^–1^, with limits of detection and quantification calculated as 0.51 and 1.58 mg L^–1^, respectively.

## 3D Printed Apparatus

This section addresses issues related to different focal distances and ambient light interference in 3D print devices.

Jarujareet et al.^38^ developed a novel 3D-printed interface that mimics a dual-beam spectrophotometer (Figure 18). This innovative tool allows users to measure absorbance (A) at various wavelengths (λ). The system can then generate absorbance vs wavelength (A vs λ) plots for colored solutions, such as permanganate and dyes. Significantly, this setup eliminates the need for separate computer processing. A dedicated smartphone app handles image analysis directly, offering a more portable and potentially more user-friendly alternative to traditional spectrophotometers.

**Figure 18:**
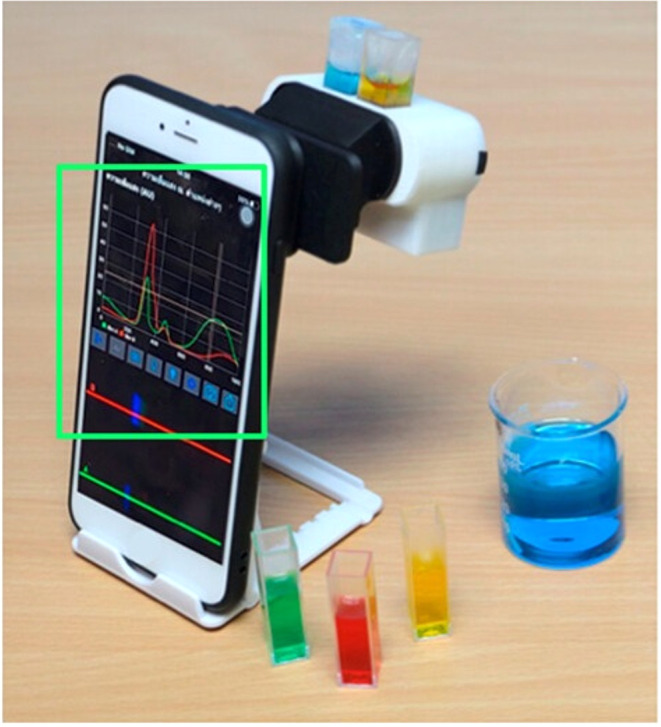
A simple dual-beam smartphone-based spectrometer attached to an iPhone 7 operated on the in-house developed iOS software. (Image reproduced from Jarujareet et al.^38^).

Pap^107^ designed a 3D apparatus that incorporated an LED, a CD serving as a diffraction grating, and either a camera or smartphone as a detector (Figure 19). This setup enabled the generation of accurate plots of absorbance vs wavelength, as demonstrated show Figure 20.^107^

**Figure 19:**
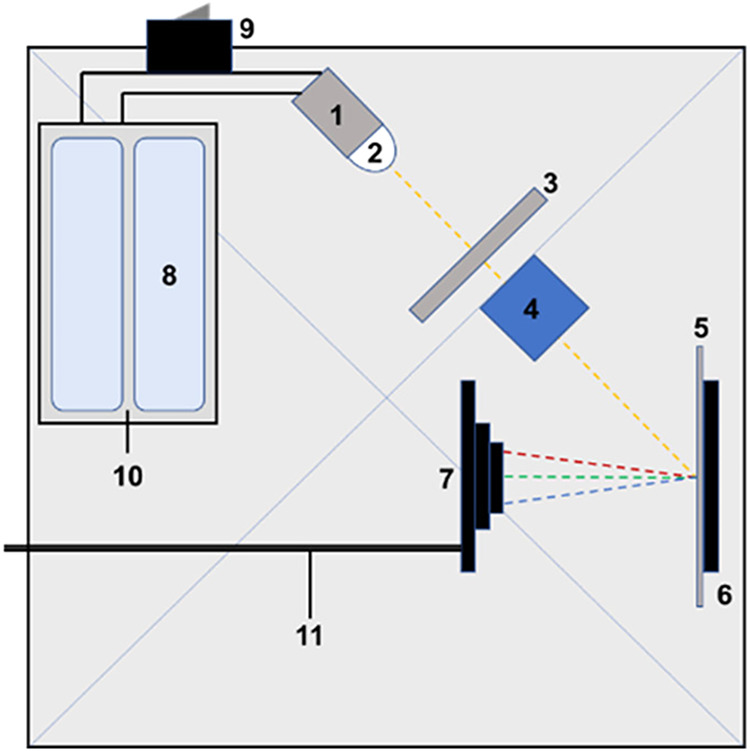
Schematic representation of the SpectroBox (top view). (1) LED light source holder, (2) LED light source, (3) slit, (4) cuvette and holder, (5) DVD-R diffraction grating, (6) grating holder, (7) web camera, (8) AA batteries, (9) switch, (10) battery holder, and (11) USB cable to the computer. (Figure reproduced from Pap et al.^107^).

**Figure 20:**
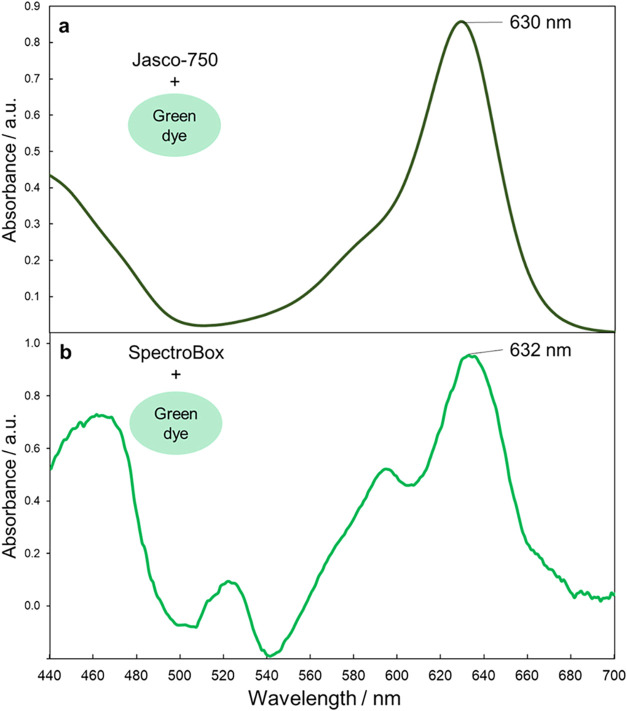
UV–vis spectrum of FD&C green no. 2 (green food dye) by (a) a Jasco V-750 UV–vis spectrophotometer and (b) data from the 3D-printed spectrometer. (Figure reproduced from Pap et al.^107^).

Bruininks & Juurlink^108^ developed a spectrometer using 3D-printed components and a smartphone as a detector. This versatile spectrometer could conduct absorption, fluorescence, and emission experiments.

Kolesnichenko et al.^109^ developed a sophisticated setup capable of conducting both absorbance and fluorescence measurements. This setup enabled absorbance studies of coumarin 151, cresyl violet, and pyridine 1, as well as fluorescence studies of rhodamine 6G and coumarin 151.

Silva et al.^110^ designed a spectrophotometer using a webcam as the detector (Figure 21). This spectrophotometer was utilized to determine Alizarin S Red absorption vs wavelength plots as a function of pH. Additionally, this spectrophotometer measured quinine fluorescence as a function of NaCl concentration.

**Figure 21:**
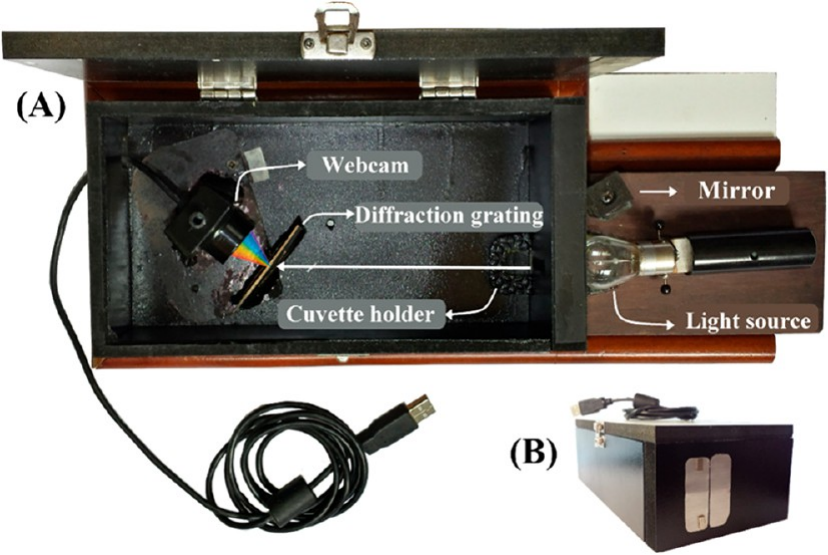
(A) View of the webcam spectrophotometer from above with all of its components: the lamp (light source), cuvette holder, diffraction grating, and webcam. (B) Device with the lid closed, where the slit can be found fixed on its front cover (Reproduced from Silva et al.^110^).

Ren et al.^111^ engineered a sophisticated apparatus employing 3D printed components (Figure 22), and this versatile setup was capable of performing a range of spectroscopic techniques, including UV–vis absorption, fluorescence, flame emission and absorption, and Raman spectroscopy. It involved the examination of various phenomena, such as the fluorescence and absorption of chlorophyll and the flame emission of elements like Li, Na, K, Sr, Ba, B, and Cu.

**Figure 22:**
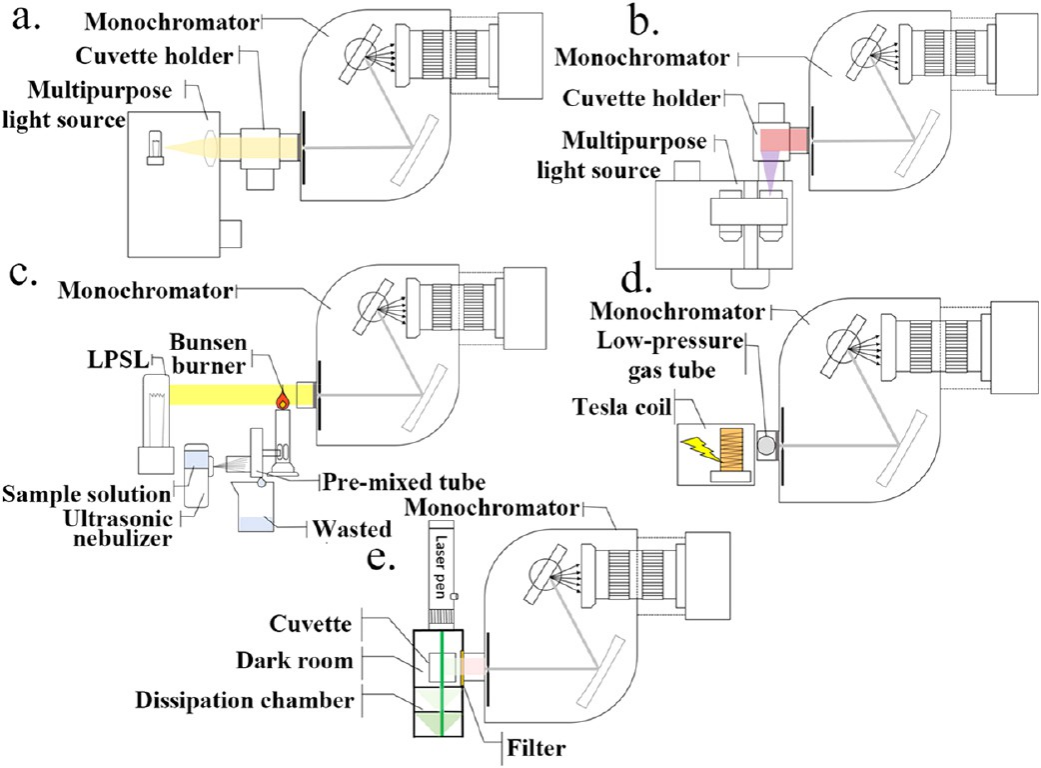
Five models switching assembly diagram of the apparatus developed by Ren et al.^111^ (a) UV–vis absorption model, (b) fluorescence emission model, (c) flame atomic emission/absorption model, (d) glow discharge model, and (e) Raman scattering model. (Reproduced from Ren et al.^111^).

Cokley et al.^112^ developed a 3D printed apparatus in which the smartphone’s camera served as the detector. This apparatus can perform both absorbance and fluorescence measurements. Studies on the absorbance of dyes and the fluorescence of fluorescein were carried out using this apparatus.

## Flame Emission

The concentration of sodium in coconut water and seawater was determined using flame emission tests analyzed through digital imaging. Flame emission images were processed by cropping with GIMP, followed by extraction of RGB values using the R programming language. The R package EBImage was utilized to subtract background signals from the flame images. This approach demonstrated a linear detection range for sodium concentrations between 20 and 160 mg L^–1^.^58^ Notably, this study was the first to apply digital image analysis to flame emission tests; however, the RGB value extraction process using R proved to be relatively complex.

Lithium concentration in the psychotropic drug lithium carbonate (Li_2_CO_3_) was determined using digital images obtained with a digital camera. In a black box, the lithium solutions were delivered into a Bunsen burner by a nebulizer. A flame image was then captured using a digital camera, and the RGB values were extracted using MATLAB.^113^

## Fluorescence

Koenig et al. published the first manuscript involving fluorescence measurements using digital images.^57^ In their study, they investigated the quenching of fluorescein by iodide. Cuvettes containing fluorescein were placed on a tablet, and an image was captured using a digital camera (as illustrated in Figure 23). This approach marked a significant step in utilizing digital imaging for fluorescence studies, but the RGB extraction was carried out using MATLAB, making it quite complicated.

**Figure 23:**
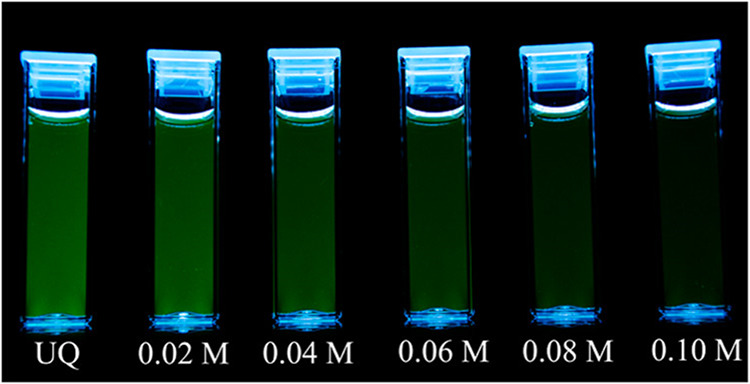
Quenching image: image of fluorescein samples with increasing iodide concentrations from left to right. UQ is the unquenched sample, and the values below each cuvette are the iodide concentrations. The decrease in emission intensity with increasing iodide concentration is clearly visible (Reproduced from Koenig et al.^57^).

Yang et al.^52^ synthesized a compound known as RhBN by reacting Rhodamine B with hydrazine. This compound was mixed with CTAB (hexadecyltrimethylammonium bromide). The resulting mixture fluoresces in the presence of hypochlorite when irradiated with 365 nm UV. The quantification of hypochlorite was achieved using ambient light. Photos were taken, and then *R* values were extracted from these photos using software tools such as ImageJ or Photoshop. This methodology allowed for the sensitive detection of hypochlorite levels.

Ambruso & Riley^85^ also proposed fluorescent experiments in which the fluorescence of rhodamine B, induced by a green laser with a wavelength of approximately 532 nm, was suppressed by Alizarin S Red. Jeong et al.^114^ developed a fluorescent spectrometer using 3D-printed components and a smartphone. This spectrometer could conduct fluorescence studies involving DNA.

Hammer et al.^39^ designed a straightforward setup for household compounds, including quinine (tonic water), curcumin (turmeric), chlorogenic acid (eggplant), and chlorophyll (spinach). In this setup, fluorescent compounds were excited with an LED, and the emitted light was captured at a perpendicular (90°) angle by a smartphone (Figure 6). The light captured by the smartphone’s camera was decomposed into RGB values using apps such as Color Grab for Android and Color Name for iPhone. The experiments revealed the fluorescence intensity as a function of concentration and the quenching of quinine due to varying NaCl concentrations.^39^

## Titrations

In acid–base titrations, both visual indicators and pH meters have traditionally been used to monitor the progression of the titration. However, there are studies that have explored the use of smartphone apps to monitor RGB values as a function of the volumes of titrant added, as illustrated in Figure 24. This approach offers a more modern and potentially more convenient way to track and analyze titration processes.^115^

**Figure 24:**
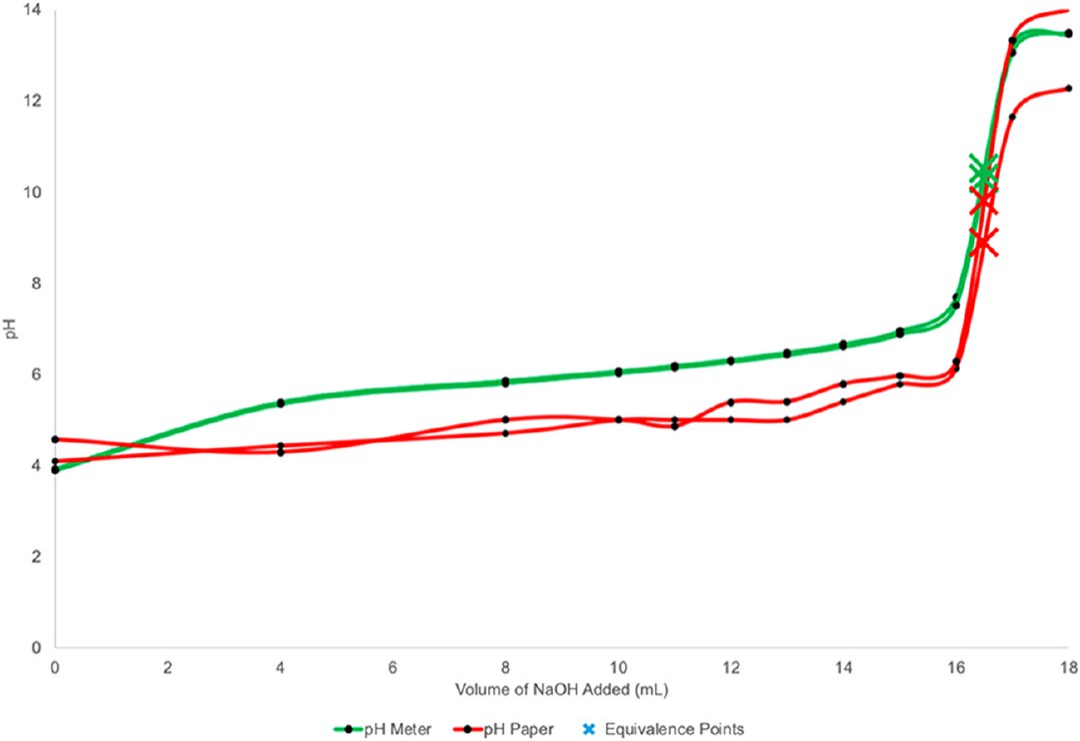
Example of acetic acid titration curves of commercial vinegar using 1.00 M NaOH. In red, the titration curves were recorded using universal pH paper and the Smart Paper Reader app. In green, the titration curves were recorded using the pH meter. (Equivalence points are represented by X and determined using the bigger pH jump.) Figure reproduced from Li et al.^115^

Smartphones have also introduced innovative teaching methods in titration classes. For example, there is an app called ″Titration ColorDarts (TCD)″ that turns titration into a competitive game. This app is designed to determine the end point of the titration and score the results on a scale from 1 to 10. It adds an element of gamification to the learning process, making titration classes more engaging and interactive.^116^

Titrations have traditionally been carried out using visual indicators, but this approach can pose challenges for students with low vision or color blindness. However, the development of the Titration ColorCam (TCC) app has made the process more accessible. TCC records and converts color information into beep sounds and vibration pulses generated by the smartphone. This enables users, including those with visual impairments, to be informed before and upon reaching the end point of the titration, making the process more inclusive.^117^

Volmer et al.^81^ developed a spectrophotometric method for analyzing red wine using images of 96-well plates. After the addition of each volume of titrant (NaOH), a small aliquot was placed in a 96-well plate, and an image was obtained using a flatbed scanner. RGB values were extracted using the ReadPlate software, and the end point was determined using the first derivative method.

Larkin et al.^118^ demonstrated the use of RGB analysis to monitor gravimetric titrations by tracking color changes of indicators. The RGB values were measured in real time using a smartphone application, allowing for direct and convenient data acquisition.

During the COVID-19 pandemic, an at-home experiment was developed to teach key concepts of acid–base equilibrium, buffer capacity, and titration curves using readily available household materials such as vinegar, cabbage extract, and sodium carbonate. The experiment required no specialized equipment, and pH changes were monitored through color variations captured with a smartphone app, which measured corresponding RGB values.^119^

Wu et al.^120^ developed a smartphone application designed to perform titrations at home. In this setup, the sample was placed in a transparent cup, and the titrant was gradually added while the color change was recorded using the smartphone’s camera positioned at a fixed distance. The app then automatically generated the titration curve based on the recorded data

## Physical Chemistry Experiments

Numerous physical and chemical experiments have been conducted utilizing smartphones. These include single-particle fluorescence microscopy,^121^ measuring detergency,^28^ measuring amylase activity,^122^ analyzing amylase,^123^ evaluating corrosion rates,^124^ and studying swelling.^125^ This section provides insights into the diverse applications of smartphones within physical chemistry laboratories.

### Determination of Equilibrium Constants (K)

The binding constant and stoichiometry ratio for the formation of the Fe^2+^ o-phenanthroline complex were determined using digital images captured with a smartphone.^67^ Cuvettes were placed inside a white box with fixed distances between the smartphone and the background (30 and 4 cm, respectively). The images were edited using GIMP 2.0, and red values were extracted using R software. These experiments were conducted as at-home activities during the COVID-19 pandemic^67^

Additionally, the pKa1 and pKa2 values of the thymol blue indicator were determined using digital images of the indicator at various pH values. The results obtained from the digital images were consistent with literature values (p*K*_a1_ = 1.65 and p*K*_a2_ = 8.96).^126^

[Co­(H_2_O)_6_]^2+^ is pink and [CoCl_4_]^2–^ is purple. The equilibrium constant of the reaction shown in eq 4, was determined using digital images obtained using the LED illuminated box. The equilibrium constant of the reaction was determined at five temperatucubic NaCl crystals, asres (25, 41, 52, 60, and 70 °C).^127^ Then, Δ*H*°, Δ*S*°, and Δ*G*° values of a colorimetric reaction were determined. The RGB values were extracted using ImageJ or directly measured using the Photometrix app.

7 $${\left[\mathrm{Co}{\left(H_{2}O\right)}_{6}\right]}^{2+}+4{\mathrm{Cl}}^{-}⇄{\left[{\mathrm{CoCl}}_{4}\right]}^{2-}+6H_{2}O$$

### Measuring Contact Angle

The contact angle may be determined using digital images obtained using smartphones^128,129^ and USB microscopes.^130^

### The Job’s Method

When a new reaction is explored or a new product is obtained in a chemical synthesis, defining the reaction stoichiometry is of primary importance,^131,132^ Olasz et al. determined the stoichiometry of precipitation reactions using the Job’s method. It was carried out using the height of precipitate columns in test tubes. Digital images of test tubes were obtained using a smartphone and the height of the columns were determined using ImageJ.^133^

### Kinect Studies

Crystal violet, a vividly violet-colored triphenylmethane dye, reacts with hydroxide ions in an aqueous solution, resulting in the formation of a colorless compound. This chemical reaction have been employed as a laboratory exercise for the experimental determination of a rate law.^134^ Knutson et al. modified this experiment, utilizing a smartphone in lieu of a spectrophotometer (see Figure 2).^35^ Green values were directly converted into an analytical signal using a dedicated app called ″color picker.″ This adaptation likely made the experiment more accessible and cost-effective.

During the COVID-19 pandemic, a unique at-home activity was proposed to determine the reaction order and kinetic parameters for the reaction between Allura red (a food dye) and hypochlorite.^135^

Cochran et al.^136^ investigated the kinetics of substrate consumption (starch) and product formation (glucose) during the enzymatic hydrolysis of starch by α-amylase. Starch concentration was determined using the starch–iodine complex, while glucose levels were measured with Benedict’s reagent. The method was effective over concentration ranges of 0.05–0.35 mg L^–1^ for starch and 0.1–0.5 mg L^–1^ for glucose. Standard and sample solutions were dispensed into a 96-well plate, and images were acquired using a flatbed scanner. RGB values were subsequently extracted and analyzed using custom software developed by the authors.

Similarly, during the COVID-19 pandemic, Barton et al.^95^ designed an at-home biochemistry lab experiment for Michaelis–Menten analysis. This experiment focused on studying tyrosinase from a crude banana extract and its inhibition by the sodium benzoate inhibitor. Such at-home experiments provided students with valuable insights into biochemistry concepts and enzyme kinetics.

Wink et al.^137^ described a student-friendly kinetics experiment that tracks the reaction between coumarin-102 and sodium hydroxide by monitoring fluorescence loss under near-UV LED illumination. Multiple cuvettes are imaged simultaneously with a smartphone, and ImageJ is used to analyze fluorescence decay. Students determine reaction orders by studying fluorescence changes over time and varying NaOH concentrations. The experiment is cost-effective, engaging, and aligns with evidence-centered design by integrating chemistry concepts with mathematical reasoning.

### Adsorption Process

In a study demonstrating the removal of methylene blue and detergent from an aqueous solution using rice husk as the adsorbent, quantification of the methylene blue removal was carried out using a smartphone.^138^ Red values were directly extracted from the samples using a dedicated app, allowing for the quantification of methylene blue in a range of 1–10 mg L^–1^. Furthermore, during the COVID-19 pandemic.

Al-Soufi et al.^65^ developed an at-home laboratory experiment where food dyes were prepared through serial dilution, and the adsorption process was measured using the Color Picker app. A cup containing water had its RGB values measured alongside standards and samples, simulating the operation of a double-beam spectrophotometer (Figure 25). These at-home experiments were designed to make scientific exploration accessible even when traditional laboratory settings were not available.

**Figure 25:**
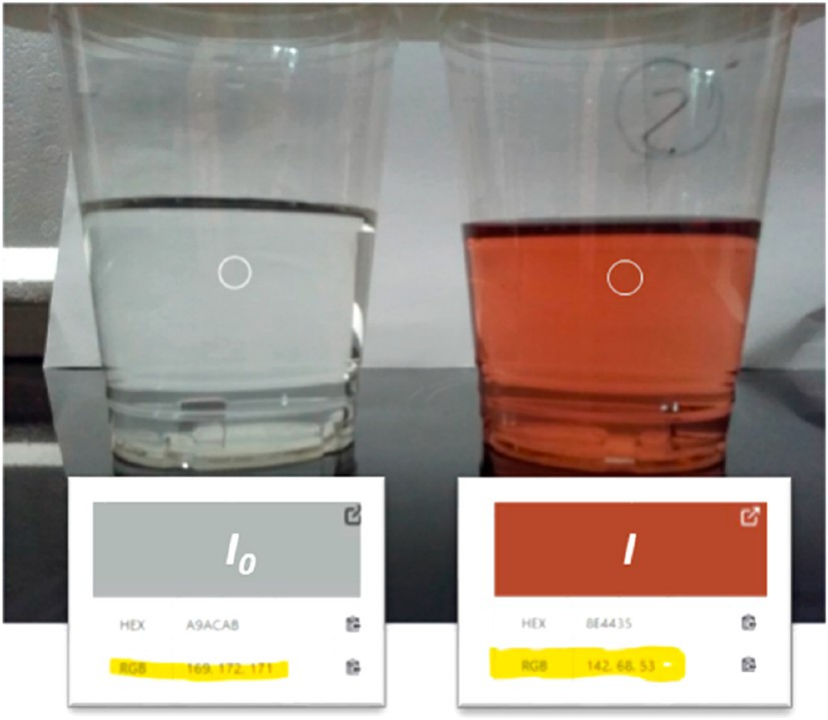
A color picker application returns the RGB intensity values in selected regions (white circles) of reference and sample cuvettes (Figure reproduced from Al-Soufi et al.^65^).

### Measurement of Liquid Diffusivities

Concentrations of a colored species in a clear liquid were assessed concerning their position and time to estimate binary diffusivity coefficients, as per Fick’s first and second laws. This experiment involved placing samples in test tubes and capturing digital images in ambient light. A laptop displaying a white screen provided the background, and a smartphone captured the images. RGB values were then extracted from the images with ImageJ.^139^ This methodology allowed for the exploration of diffusion processes using simple and accessible equipment.

### Polymer Degradation

In Knutson et al.^36^ experiments, a yellow dye, (λ_max_) of 425 nm, was introduced into polymer samples. The polymer samples were then exposed to an aqueous sodium hydroxide solution, causing them to degrade and release the dye. The concentration of the yellow dye in the solution served as an indicator of polymer degradation. This concentration was measured using the method described by Penn and his co-workers,^35,101^ as illustrated in Figure 3. In this process, the blue channel, representing the complementary color to yellow, was found to exhibit more significant variations as a function of dye concentration, making it a suitable choice for quantification.

### Infrared Cameras

Infrared Cameras and smartphones equipped with infrared capabilities were not as prevalent in the past. However, in recent years, these devices have become cheaper than in the past. Smartphones featuring infrared cameras were employed to observe temperature variations during various processes, including the crystallization of supersaturated NaOAc, the vaporization of water, KCl solution, and saturated KCl, as well as the mixing of ethanol with water and cyclohexane.^140^ These smartphones with infrared cameras were also utilized to investigate heat transfer phenomena, such as the transfer of heat between a hot block and a cold block (as depicted in Figure 26).^141^

**Figure 26:**
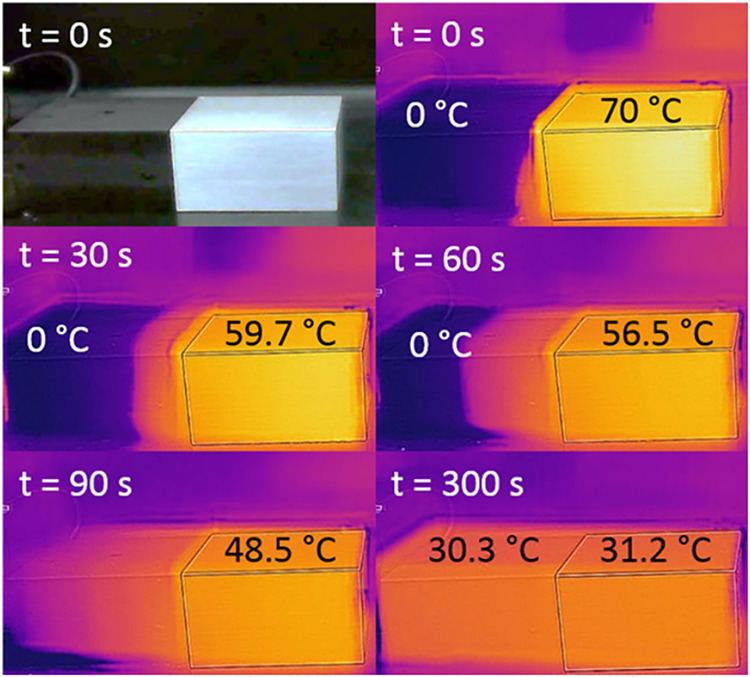
Two aluminum blocks depicted in the top-left visible-light image were cooled to 0 °C (black/left block) and heated to 70 °C (white/right block), respectively. The blocks were placed in contact and imaged every 30 s for 5 min to visualize transfer of heat between the two blocks (Figure reproduced from Green et al.^141^).

### Catalysis

The catalytic reduction of 4-Nitrophenol by NaBH_4_ in the presence of silver nanoparticles was studied.^142^ The 4-nitrophenol reduction results in color change from yellow-green to colorless. Its concentration was measured using gray values extracted from digital images obtained with a smartphone under a homemade control lightbox. Images obtained using the smartphone were loaded onto a computer and its RGB-values were extracted using ImageJ.

### Smartphones as Microscopes

Smartphone cameras have been repurposed as microscopes, using a simple setup that includes a glass bead serving as a lens, held in place by a plastic clip.^143^ his makeshift microscope enabled the observation of dissolution and precipitation phenomena. For example, it revealed that table salt (Figure 27a) typically has a cubic shape, whereas sea salt does not exhibit this feature (Figure 27b). However, through a recrystallization process, sea salt was induced to display a cubic shape (Figure 27c).

**Figure 27:**
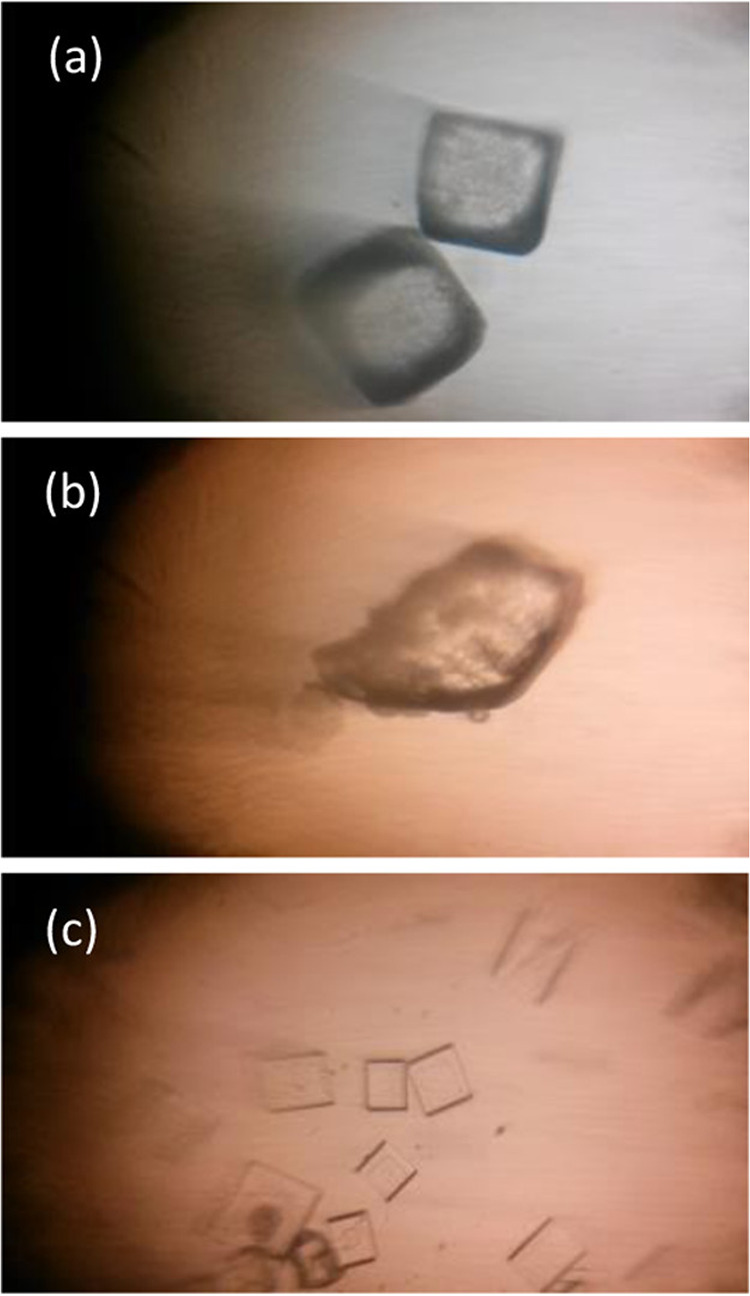
Static micrographs of (a) Morton table salt, (b) Alessi Mediterranean Sea salt, and (c) recrystallized sea salt. (Figure reproduced from Lumetta & Arcia^143^).

Smartphone microscopes were utilized to explore the reaction involving one drop of CoCl_2_ and eight drops of NaOH (as illustrated in Figure 28).^144^ This experiment revealed the formation of pink Co­(OH)_2_, as described by eq 8, and the blue Co­(OH)Cl products, as indicated in eq 8. The specific product formed was found to depend on the concentration of NaOH used in the reaction. Additionally, this setup allowed for the observation of cubic NaCl crystals, as depicted in Figure 21.

8 $${\mathrm{CoCl}}_{2}+2\mathrm{NaOH}⇄\mathrm{Co}{\left(\mathrm{OH}\right)}_{2}↓\left(\mathrm{pink}\right)+2\mathrm{NaCl}$$

9 $${\mathrm{CoCl}}_{2}+\mathrm{NaOH}⇄\mathrm{Co}\left(\mathrm{OH}\right)\mathrm{Cl}↓\left(\mathrm{blue}\right)+\mathrm{NaCl}$$

**Figure 28:**
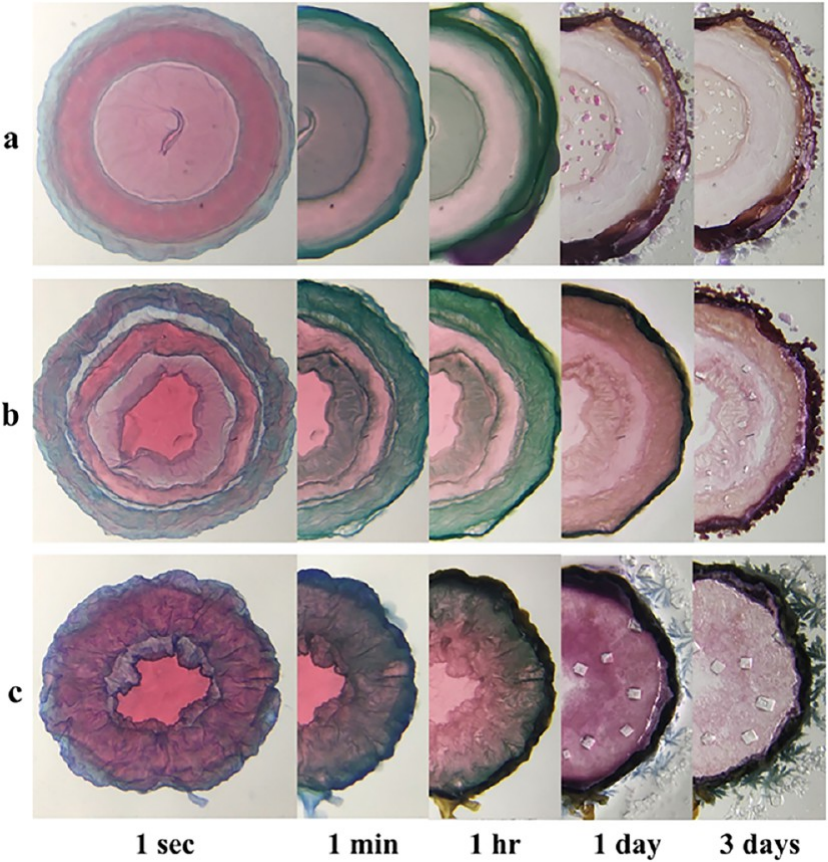
Three examples of cobalt chloride reacted with sodium hydroxide: 1 drop of 2 M cobalt chloride solution was dropped into 8 drop of (a) 1 M, (b) 0.5 M, and (c) 0.25 M sodium hydroxide solution; for the sake of comparison, only the right half of the precipitate is displayed, except for the first picture. Photograph reproduced with permission (Figure reproduced from Ling et al.^144^).

## Conclusions

The integration of digital imaging technology into laboratory experiments has significantly expanded the possibilities for both scientific research and education. By utilizing widely available devices such as smartphones, these methods have democratized access to advanced analytical techniques, making them viable for institutions with limited resources. The COVID-19 pandemic further highlighted the potential of home-based laboratory setups, demonstrating the practicality and effectiveness of digital images for quantitative analysis.

The versatility of digital imaging has been showcased across a broad spectrum of applications, ranging from flame emission spectroscopy to fluorescence analysis. Transitioning from traditional spectrophotometric methods to RGB value extraction has proven not only cost-effective but also highly engaging for students. This approach promotes deeper conceptual understanding by encouraging learners to explore scientific phenomena using familiar, everyday technology.

The continued development and implementation of digital imaging in laboratory settings holds substantial promise for the future of science education and research. These techniques enhance the educational experience and offer practical solutions for conducting rigorous experiments beyond conventional laboratories. As imaging technology continues to evolve, its applications in the sciences will likely grow, further enriching both educational practices and the broader field of chemical analysis.
